# Correction: Association of human breast cancer CD44^-^/CD24^-^ cells with delayed distant metastasis

**DOI:** 10.7554/eLife.109109

**Published:** 2025-09-10

**Authors:** Xinbo Qiao, Yixiao Zhang, Lisha Sun, Qingtian Ma, Jie Yang, Liping Ai, Jinqi Xue, Guanglei Chen, Hao Zhang, Ce Ji, Xi Gu, Haixin Lei, Yongliang Yang, Caigang Liu

**Keywords:** Human, Mouse

Qiao X, Zhang Y, Sun L, Ma Q, Yang J, Ai L, Xue J, Chen G, Zhang H, Ji C, Gu X, Lei H, Yang Y, Liu C. 2021. Association of human breast cancer CD44-/CD24- cells with delayed distant metastasis. *eLife* **10**:e65418. doi: 10.7554/eLife.65418.

Published 28 July 2021

The authors sincerely acknowledge that an incorrect western blot image (Figure 7C) and a mammosphere formation image (Figure 7F) were inadvertently included in Figure 7 of the originally published article. This error resulted from a mislabeled file originating from a parallel project conducted under different experimental conditions.

In our laboratory, experimental data are organized and stored according to project and experiment type. For instance, images related to sphere formation are saved in a structured folder hierarchy such as: “CSC sphere” → “MDA-MB-231” → “Ctl” or “siRHBDL2.” Because subfolders from different projects may share identical names (e.g., “Ctl”), and some image files had similar or ambiguous names, an image from a separate experiment was inadvertently saved into the current project’s folder and mistakenly used in figure preparation. Furthermore, the erroneous image had been cropped during figure processing, further masking its identity and contributing to the oversight.

In Figure 7C, the following western blot panels were mistakenly used and have been replaced with the correct ones:

MDA-MB-231 cells: α-Tubulin and Lamin B panels

MDA-MB-468 cells: Lamin B panel

In Figure 7F, the mammosphere formation images for CD44-/CD24- MDA-MB-231 cells, in the group “Ctl” and “siRHBDL2”, were incorrectly presented and have now been replaced with the appropriate images from the original experimental dataset.

Importantly, the quantitative analysis displayed in the bar graph below Figure 7F, representing mammosphere number and average diameter, was independently derived from the correct raw images obtained from three biological replicates. Therefore, the quantification and statistical results are accurate and do not require correction.

To rectify these issues and uphold the integrity of the publication, the authors have thoroughly reviewed the original data and replaced the incorrect images with the appropriate ones derived from the correct experiments. The associated source data for Figure 7C and 7D (Figure 7—Source data 1) has also been corrected.

We deeply regret this oversight and have since instituted more stringent quality control measures, including standardized file naming conventions and mandatory cross-verification steps during figure assembly, to prevent such errors in the future.

The corrected Figure 7 with updated panels C and F are shown here:

**Figure 1:**
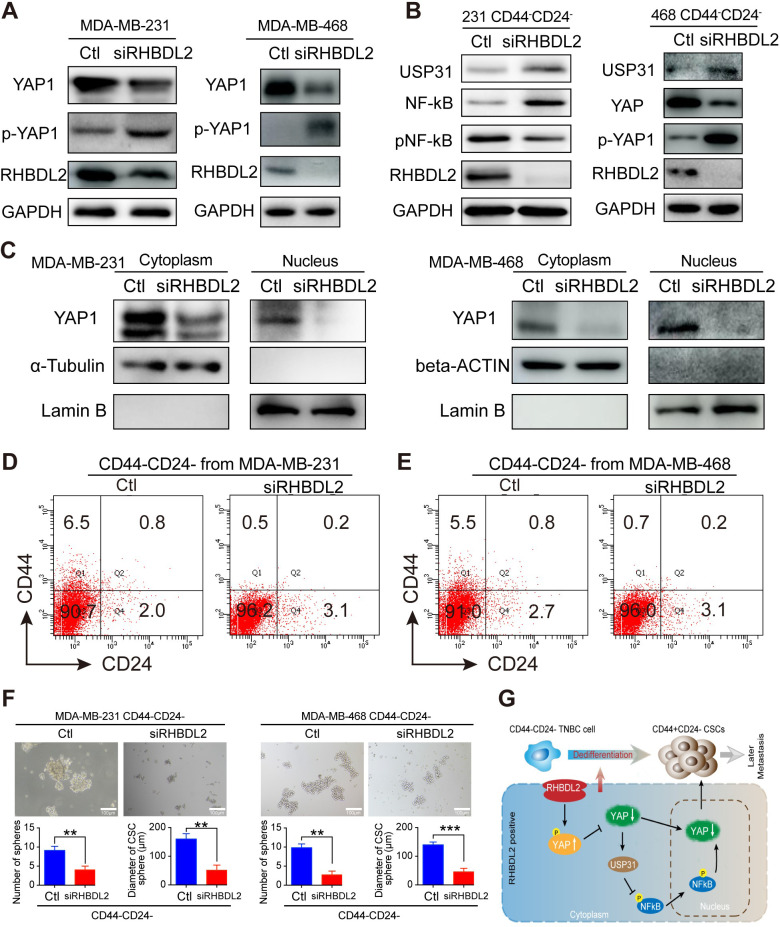


The relevant text from the legend associated with panels C and F are shown here:

**Figure 7. RHBDL2 silencing inhibits the spontaneous conversion of CD44– /CD24– cells into CSCs by attenuating the YAP1/NF-kB signaling through enhancing USP31 expression in TNBC cells.**

Western blot analysis of cytoplasmic and nuclear YAP1 protein levels in MDA-MB-231/MDA-MB-468 cells following RHBDL2 silencing indicated that RHBDL2 silencing mitigated the nuclear translocation of YAP1 in TNBC cells.

(**F**) RHBDL2 silencing attenuated mammosphere formation of CD44-/CD24- CSCs. Following transfected with the control or RHBDL2-specific siRNA, CD44-/CD24- CSCs were cultured in L15 medium for 7 days and the formed mammospheres were photoimaged (magnification x 400) and their numbers and sizes were measured in a blinded manner. Data are representative images or expressed as the mean ± SD of each group from at least three separate experiments. *p <0.05, **p <0.01, determined by Student t-test.

The originally published Figure 7 is shown for reference:

**Figure 2:**
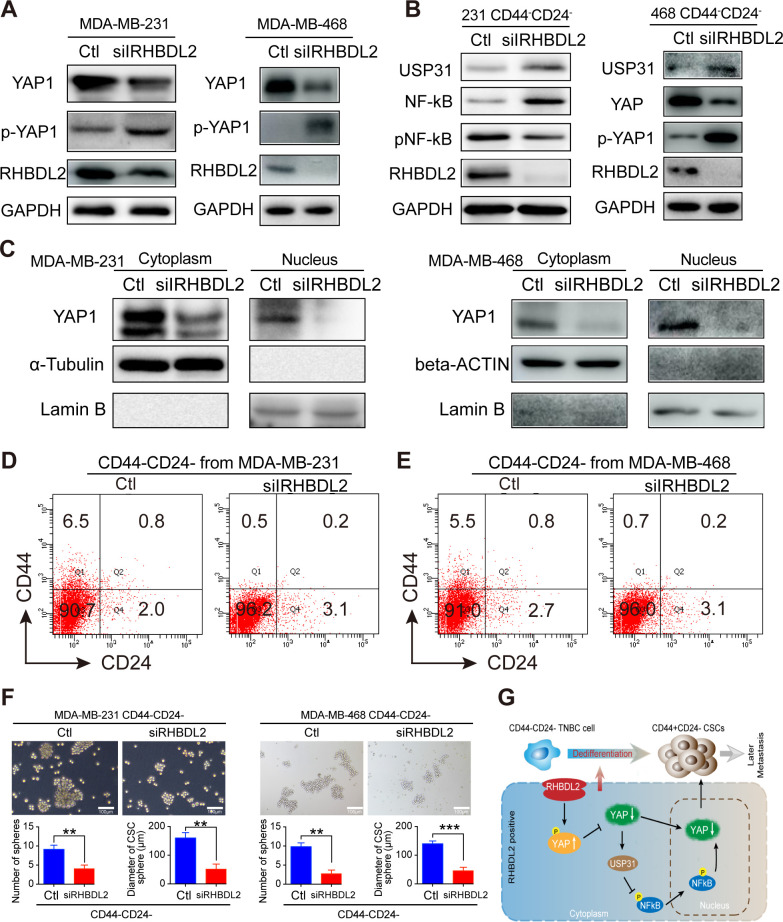


These errors have no impact on the results, interpretations, or conclusions of this study.

The article has been corrected accordingly.

