# Supplementary figures and images for: Association of human breast cancer CD44-/CD24- cells with delayed distant metastasis
Source: eLife. 2021 Jul 28;10:e65418. doi: 10.7554/eLife.65418 (PMC8346282; doi:10.7554/eLife.65418)

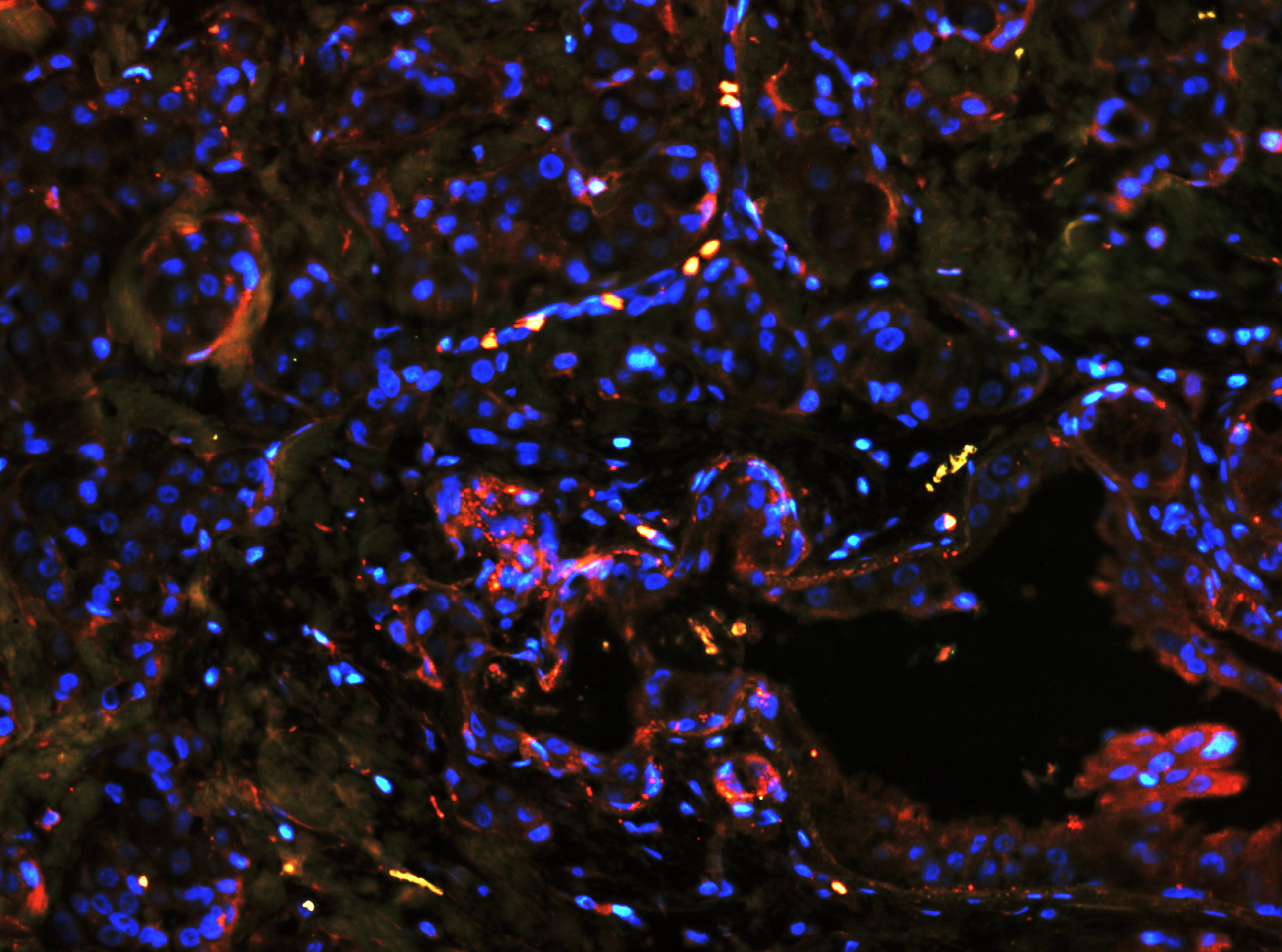

Supplement: Figure 1—figure supplement 1—source data 1. [file elife-65418-fig1-figsupp1-data1.zip › Fig 1-Supplement fig 1/A/A1.tif]

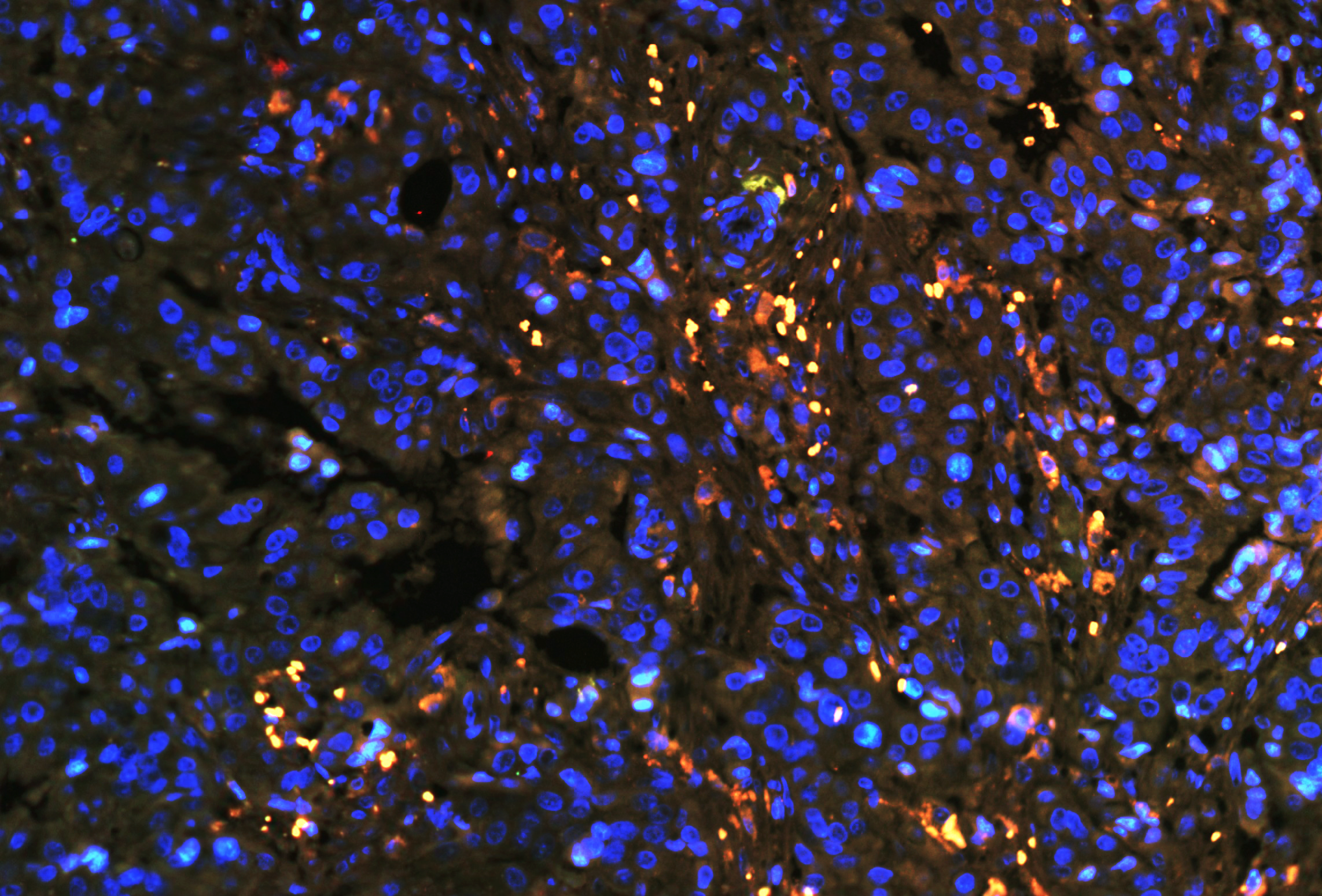

Supplement: Figure 1—figure supplement 1—source data 1. [file elife-65418-fig1-figsupp1-data1.zip › Fig 1-Supplement fig 1/A/A2.tif]

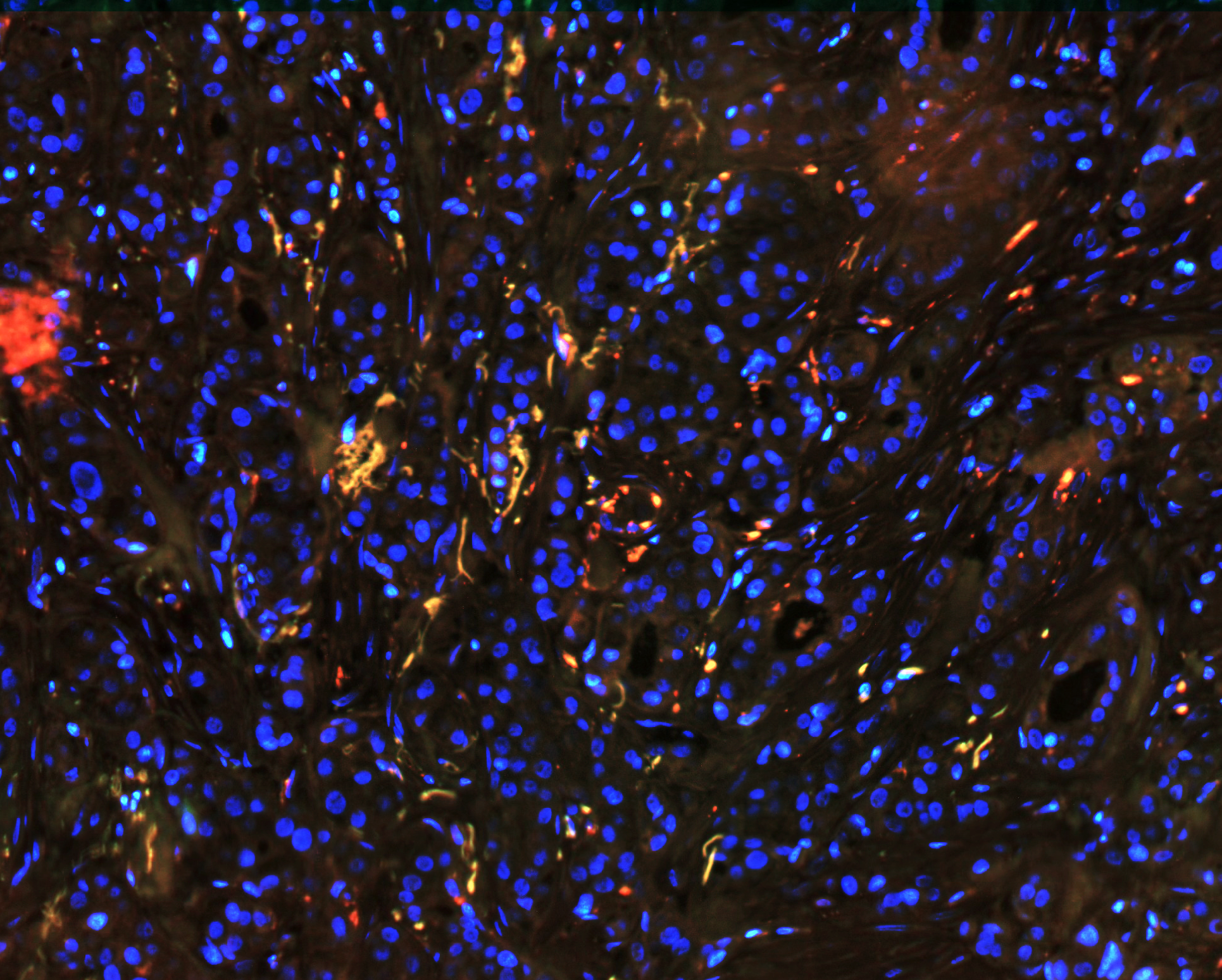

Supplement: Figure 1—figure supplement 1—source data 1. [file elife-65418-fig1-figsupp1-data1.zip › Fig 1-Supplement fig 1/B/B1.tif]

# BD FACSDiva 8.0.1

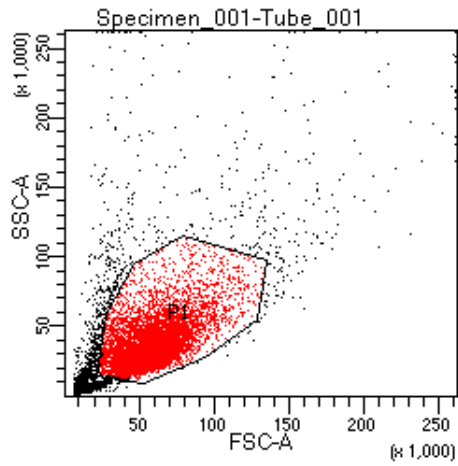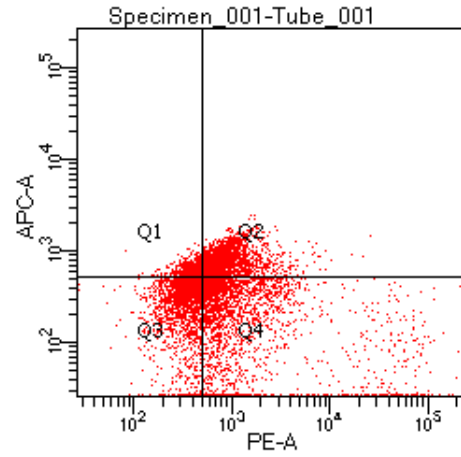

| Tube: Tube_001 |         |         |        |
|----------------|---------|---------|--------|
| Population     | #Events | %Parent | %Total |
| ■ All Events   | 10,000  | ####    | 100.0  |
| ■ P1           | 7,525   | 75.2    | 75.2   |
| ☒ Q1           | 674     | 9.0     | 6.7    |
| ☒ Q2           | 2,246   | 29.8    | 22.5   |
| ☒ Q3           | 2,104   | 28.0    | 21.0   |
| ☒ Q4           | 2,501   | 33.2    | 25.0   |

Supplement: Figure 3—source data 1. [file elife-65418-fig3-data1.zip › Fig 3/B/1.pdf]

# BD FACSDiva 8.0.1

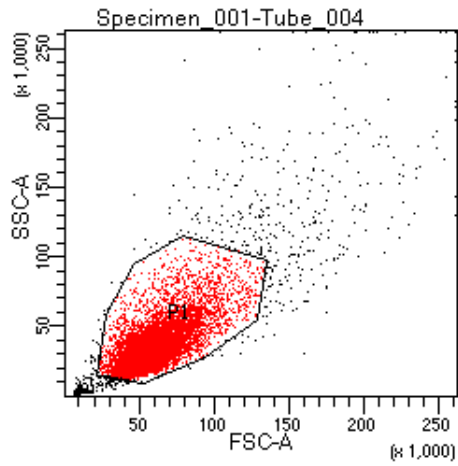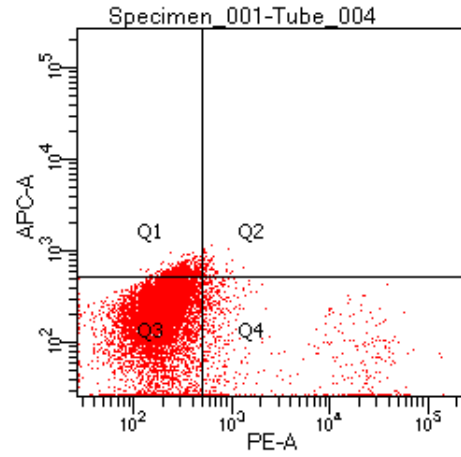

| Tube: Tube_004 |         |         |        |
|----------------|---------|---------|--------|
| Population     | #Events | %Parent | %Total |
| ■ All Events   | 10,000  | ####    | 100.0  |
| ■ P1           | 9,254   | 92.5    | 92.5   |
| ☒ Q1           | 429     | 4.6     | 4.3    |
| ☒ Q2           | 59      | 0.6     | 0.6    |
| ☒ Q3           | 8,166   | 88.2    | 81.7   |
| ☒ Q4           | 600     | 6.5     | 6.0    |

Supplement: Figure 3—source data 1. [file elife-65418-fig3-data1.zip › Fig 3/B/2.pdf]

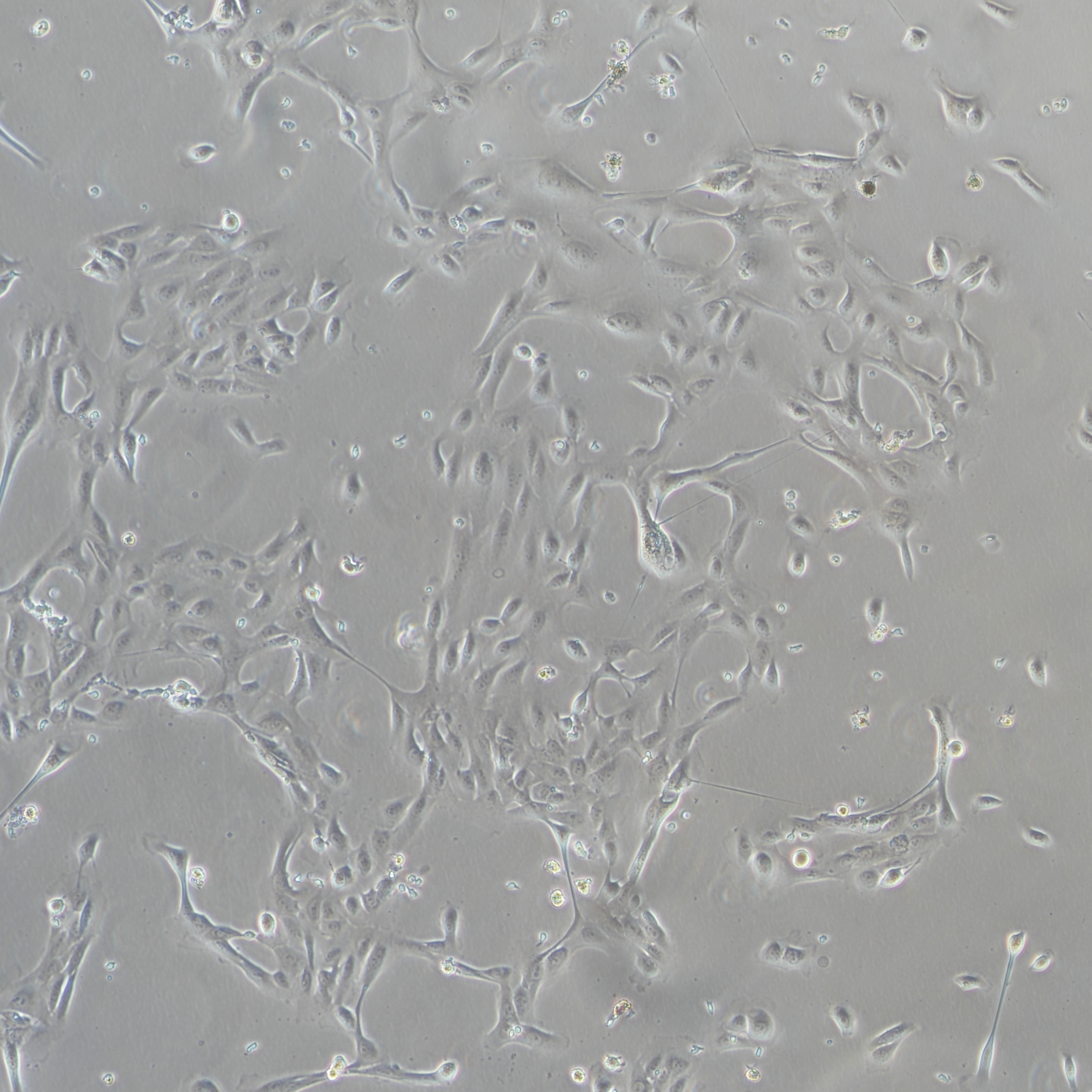

Supplement: Figure 3—figure supplement 1—source data 1. [file elife-65418-fig3-figsupp1-data1.zip › Fig 3-Supplement fig 1/A/2.tif]

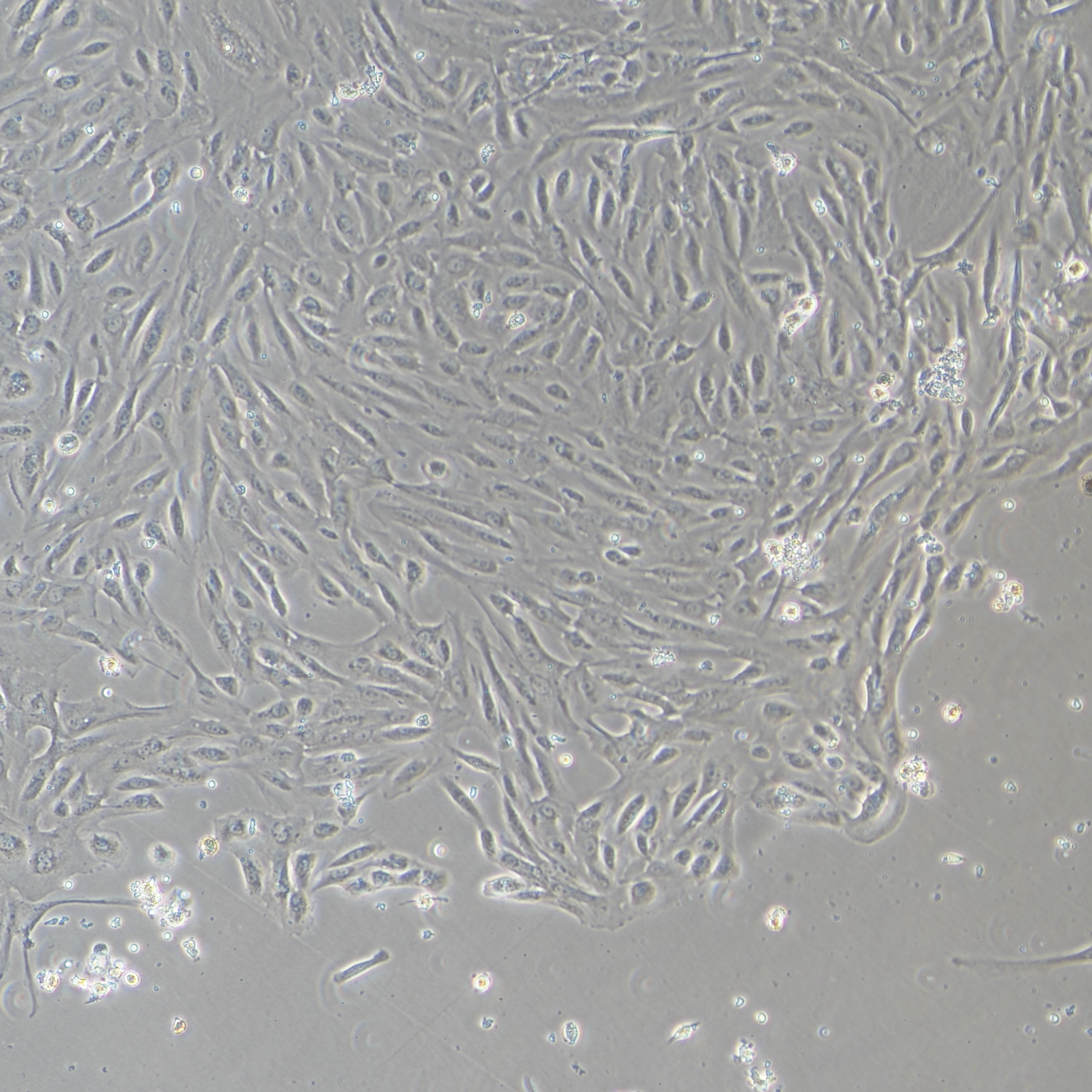

Supplement: Figure 3—figure supplement 1—source data 1. [file elife-65418-fig3-figsupp1-data1.zip › Fig 3-Supplement fig 1/A/1.tif]

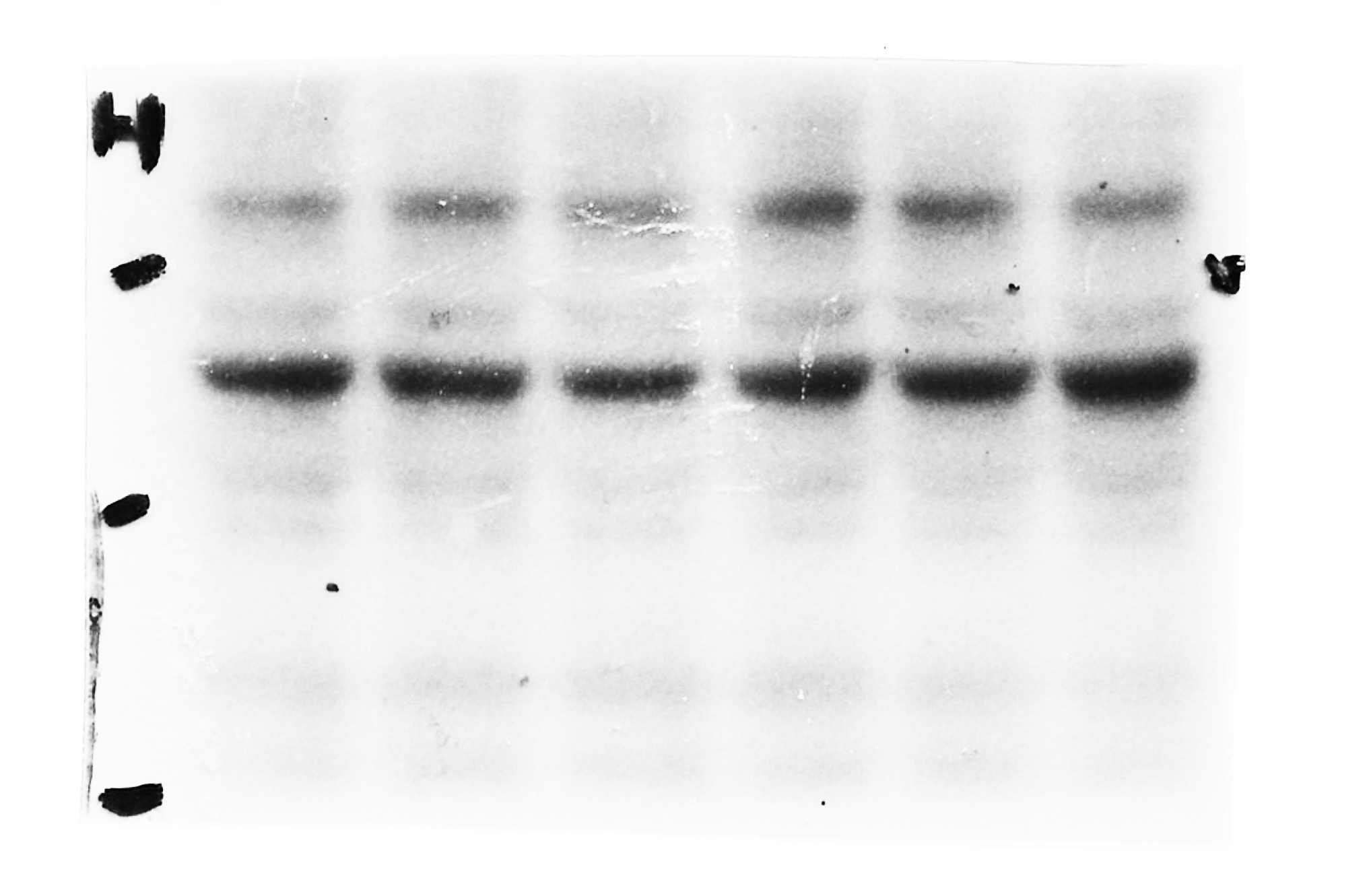

Supplement: Figure 4—source data 1. [file elife-65418-fig4-data1.zip › Fig 4/D/nanog.png]

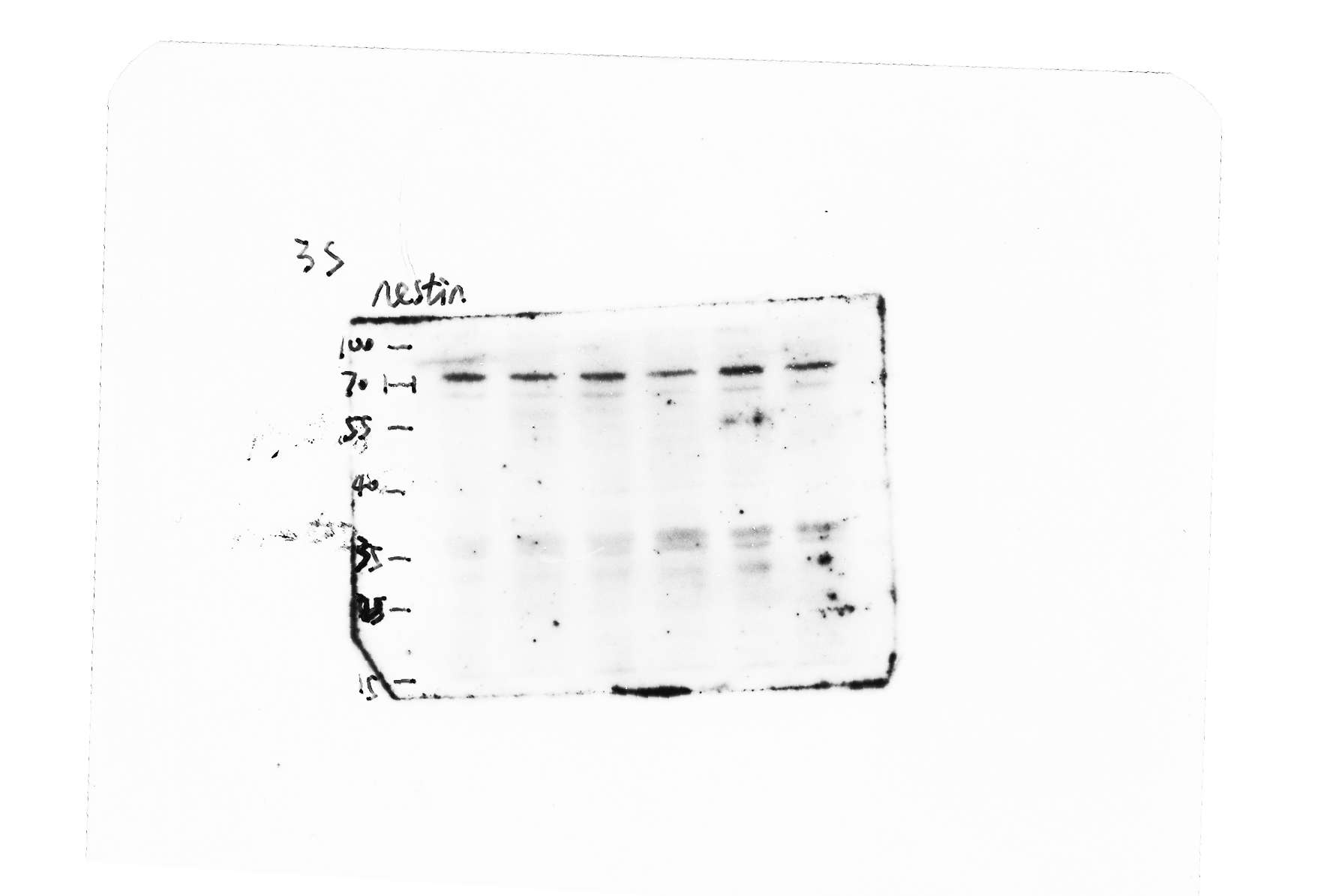

Supplement: Figure 4—source data 1. [file elife-65418-fig4-data1.zip › Fig 4/D/nestin-.png]

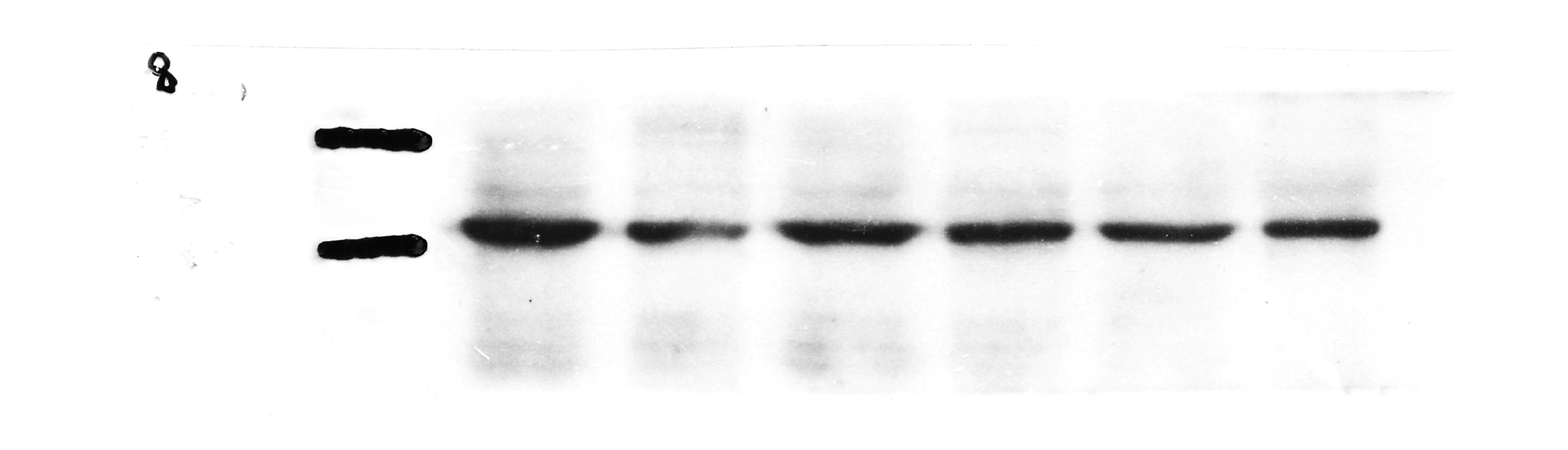

Supplement: Figure 4—source data 1. [file elife-65418-fig4-data1.zip › Fig 4/D/oct4.png]

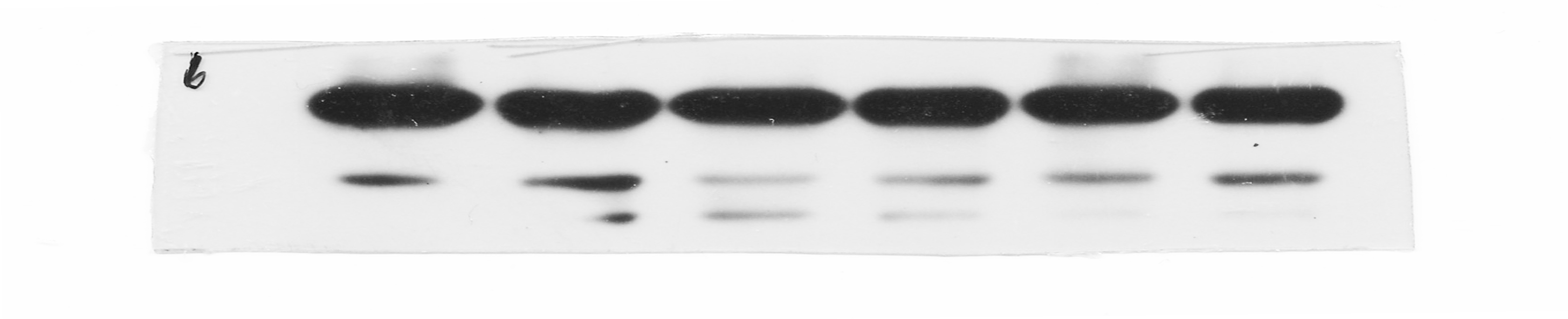

Supplement: Figure 4—source data 1. [file elife-65418-fig4-data1.zip › Fig 4/D/gapdh.png]

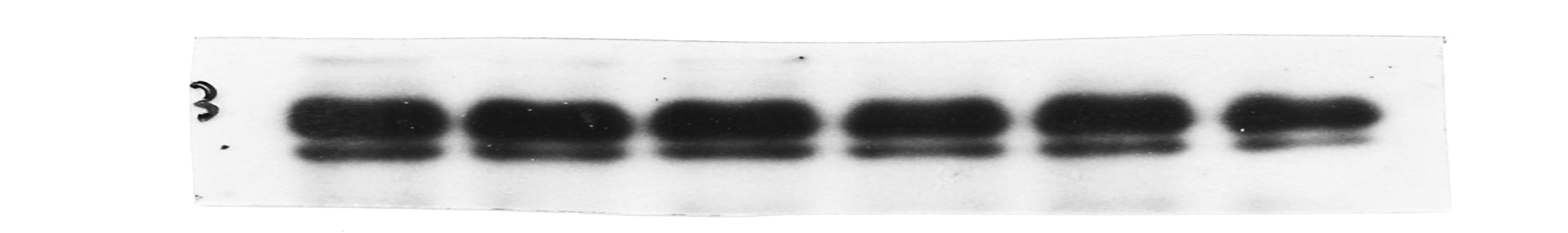

Supplement: Figure 4—source data 1. [file elife-65418-fig4-data1.zip › Fig 4/D/sox2.png]

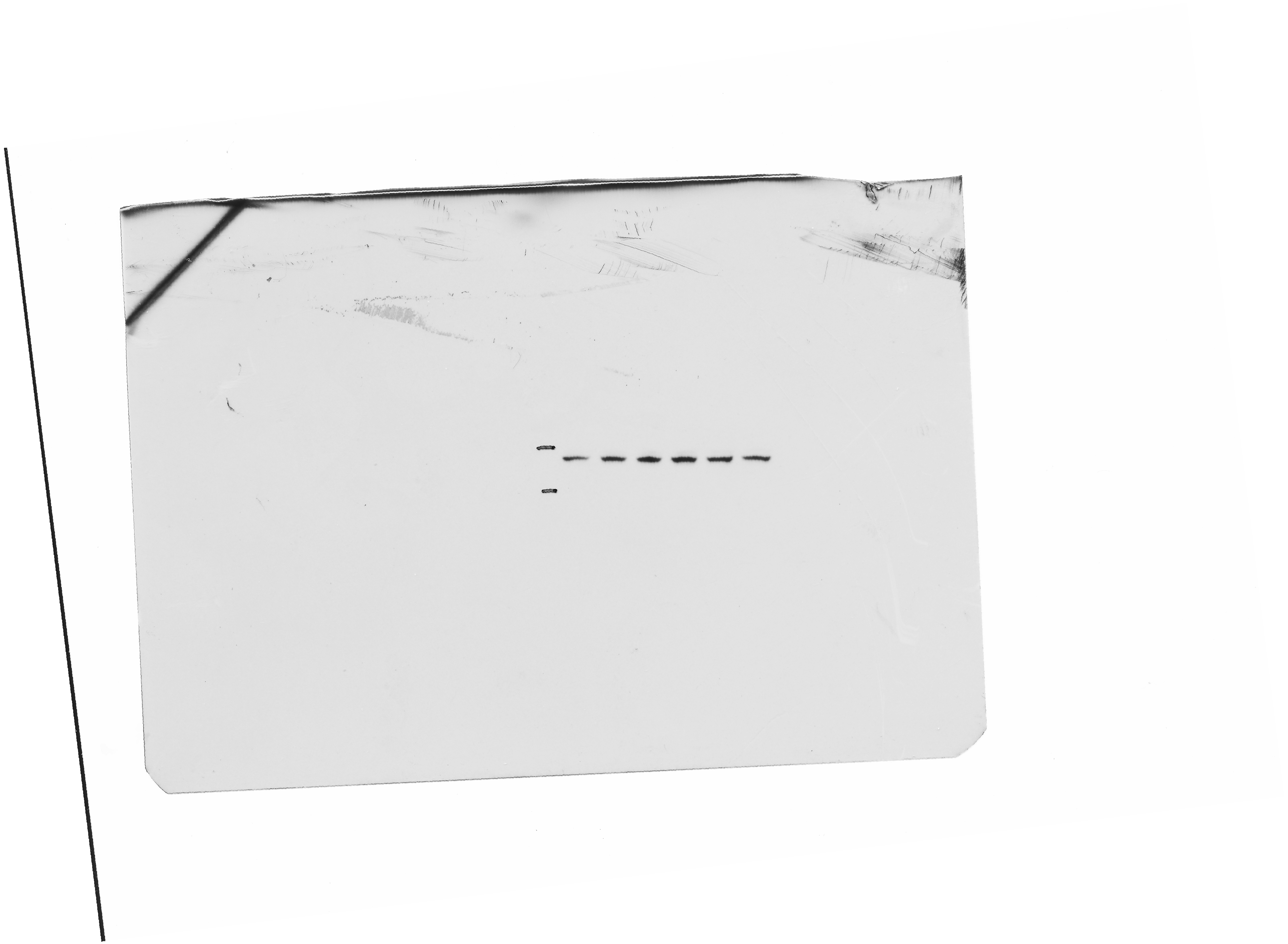

Supplement: Figure 4—source data 1. [file elife-65418-fig4-data1.zip › Fig 4/D/ABCB1.png]

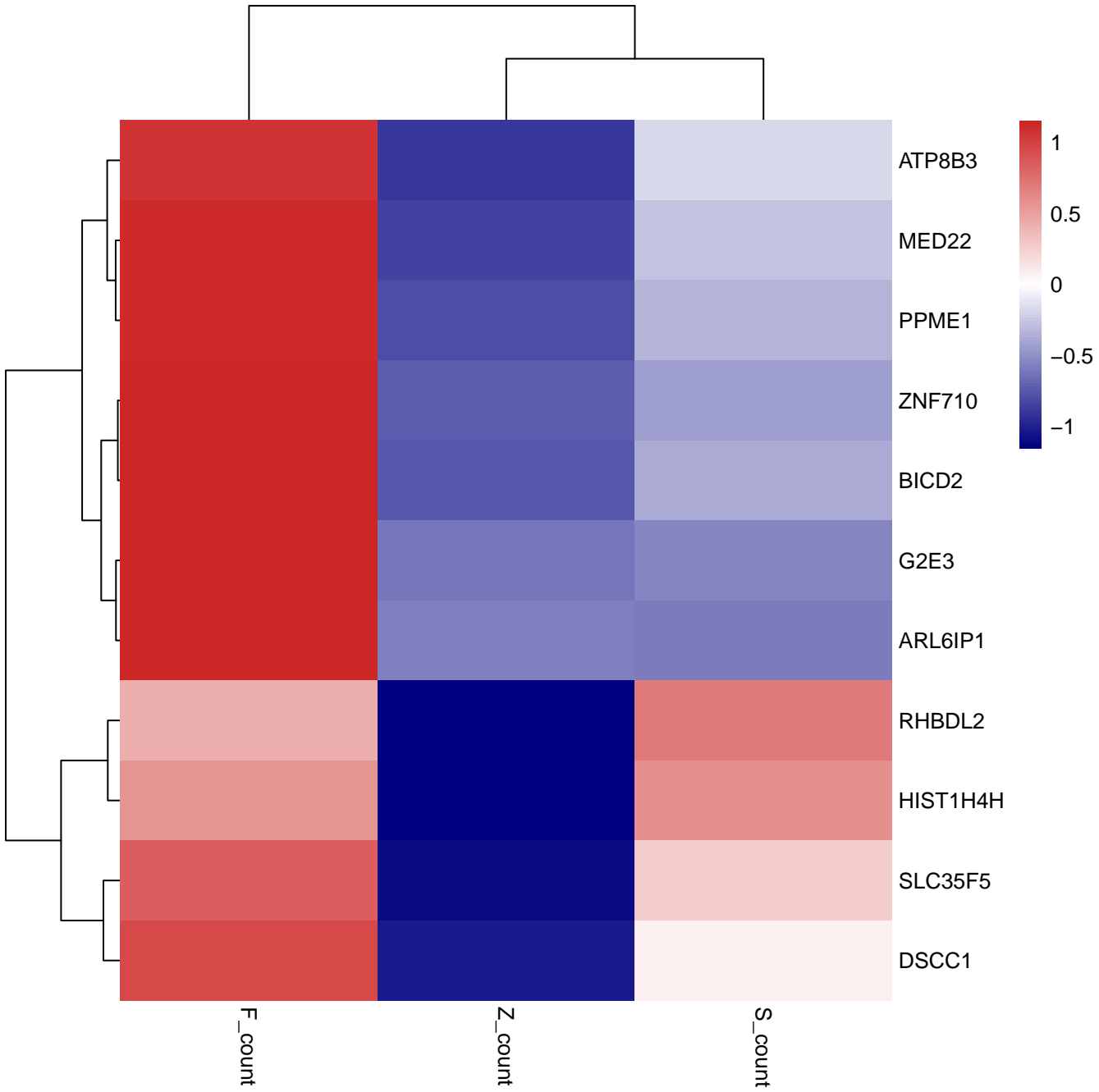

Supplement: Figure 6—source data 1. [file elife-65418-fig6-data1.zip › Fig 6/B/pheatmap.pdf]

# H4/h (208180\_s\_at)

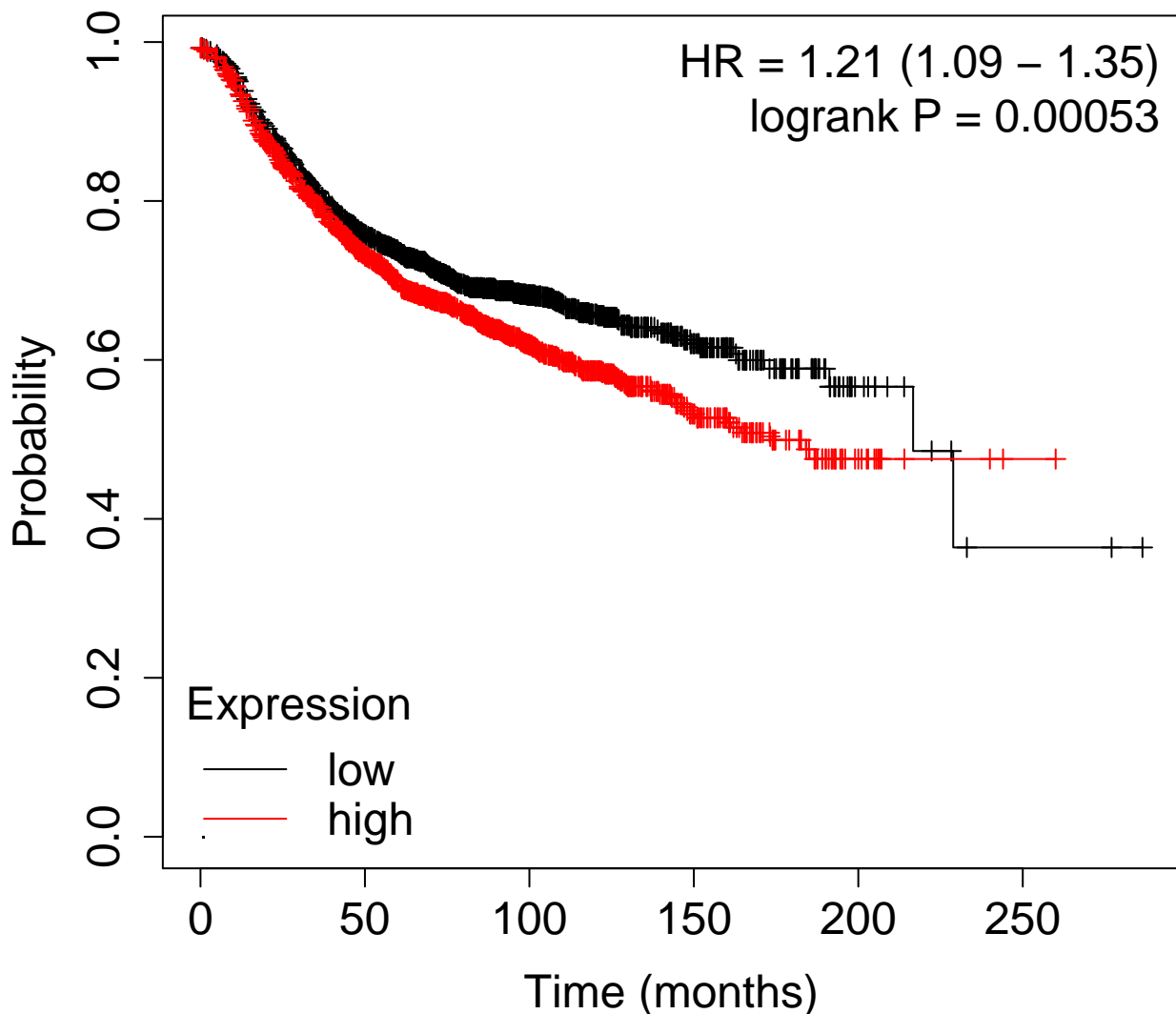

Number at risk

|      |      |      |     |     |    |   |
|------|------|------|-----|-----|----|---|
| low  | 1983 | 1304 | 554 | 126 | 13 | 2 |
| high | 1968 | 1215 | 521 | 115 | 14 | 1 |

Supplement: Figure 6—source data 1. [file elife-65418-fig6-data1.zip › Fig 6/C/KM plotter/HIST1H4H.pdf]

# ATP8B3 (239457\_at)

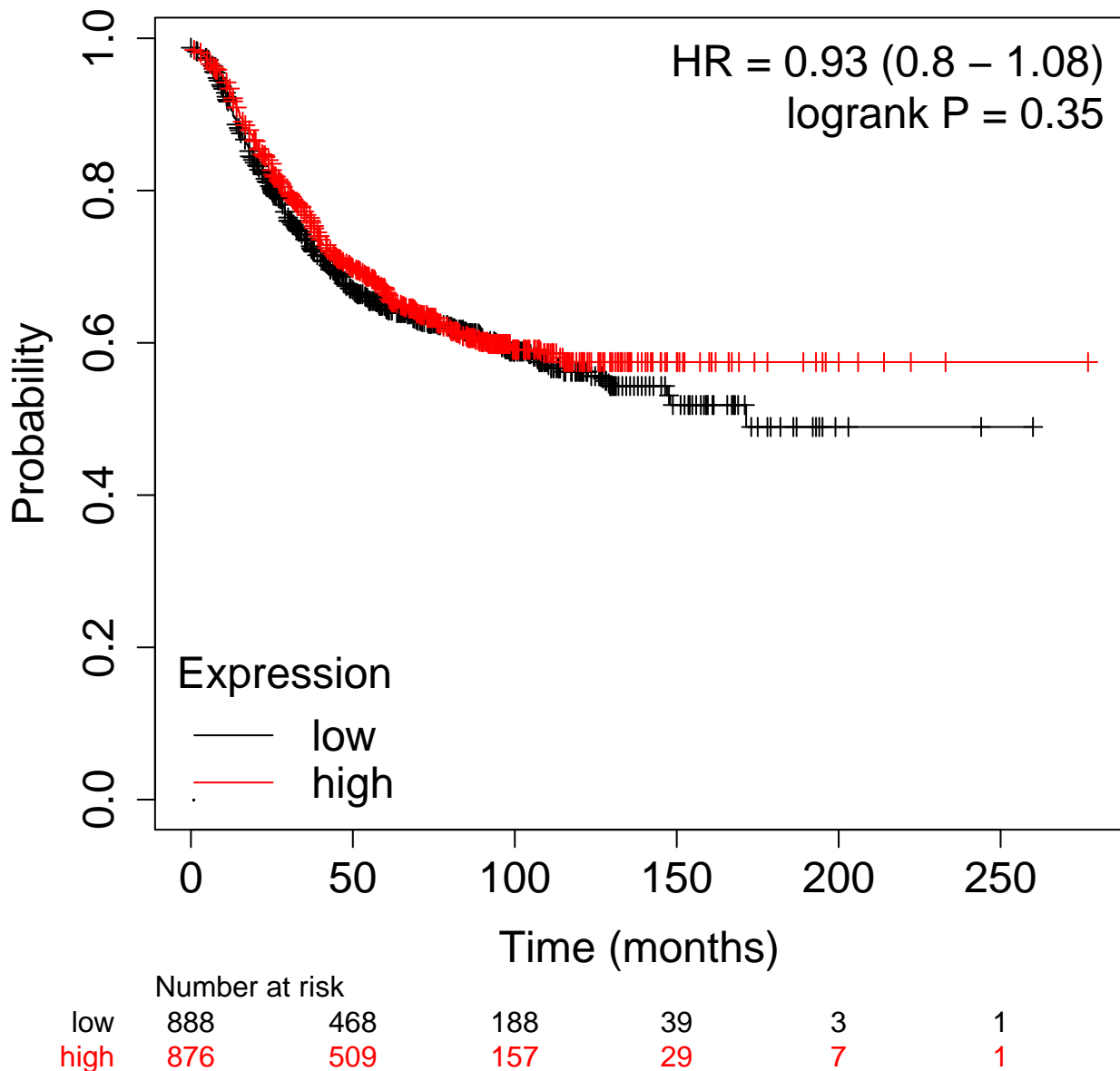

Supplement: Figure 6—source data 1. [file elife-65418-fig6-data1.zip › Fig 6/C/KM plotter/ATP8B3.pdf]

# G2E3 (223257\_at)

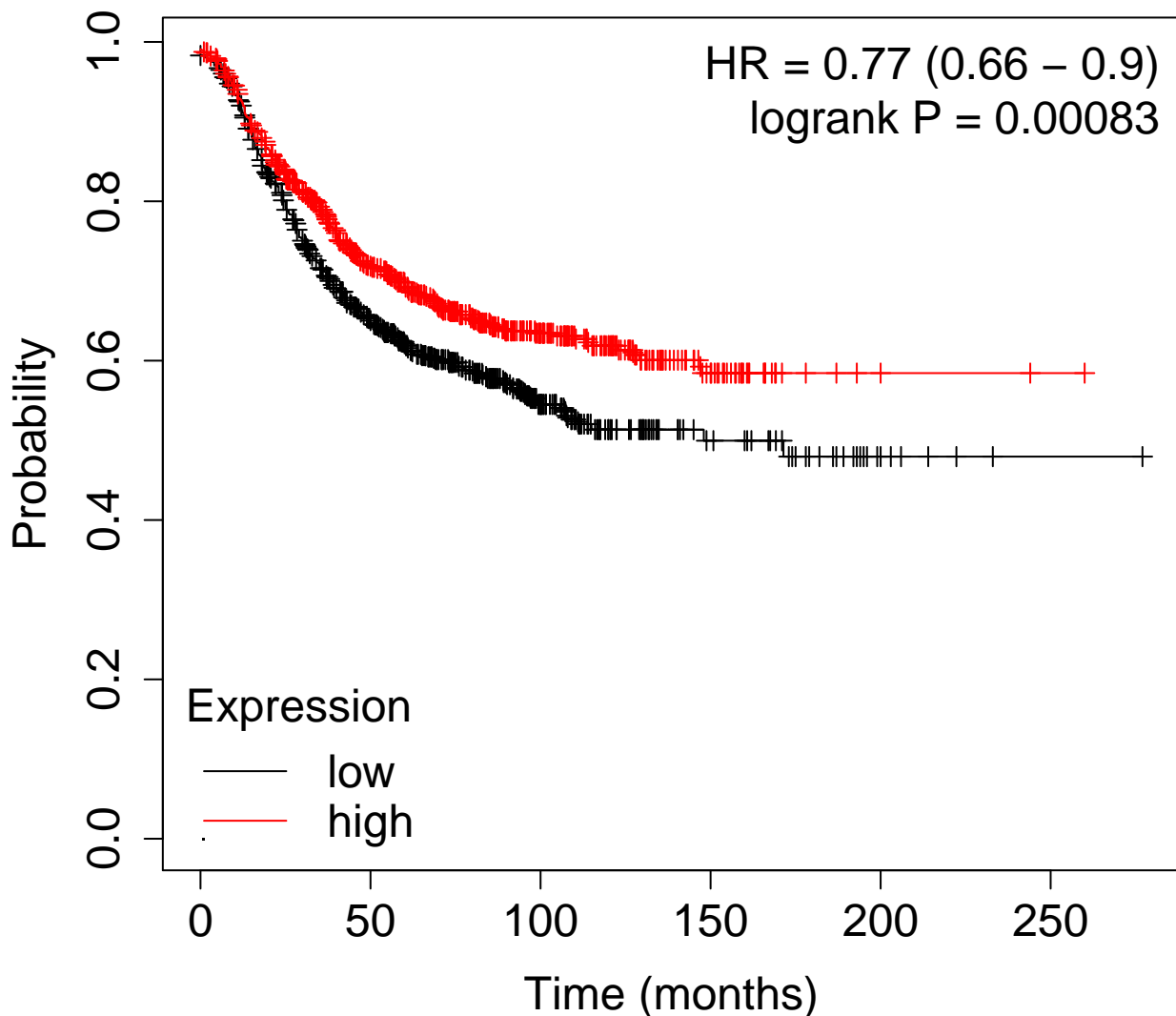

Number at risk

|      |     |     |     |    |   |   |
|------|-----|-----|-----|----|---|---|
| low  | 882 | 489 | 142 | 35 | 7 | 1 |
| high | 882 | 488 | 203 | 33 | 3 | 1 |

Supplement: Figure 6—source data 1. [file elife-65418-fig6-data1.zip › Fig 6/C/KM plotter/G2E3.pdf]

# SLC35F5 (225872\_at)

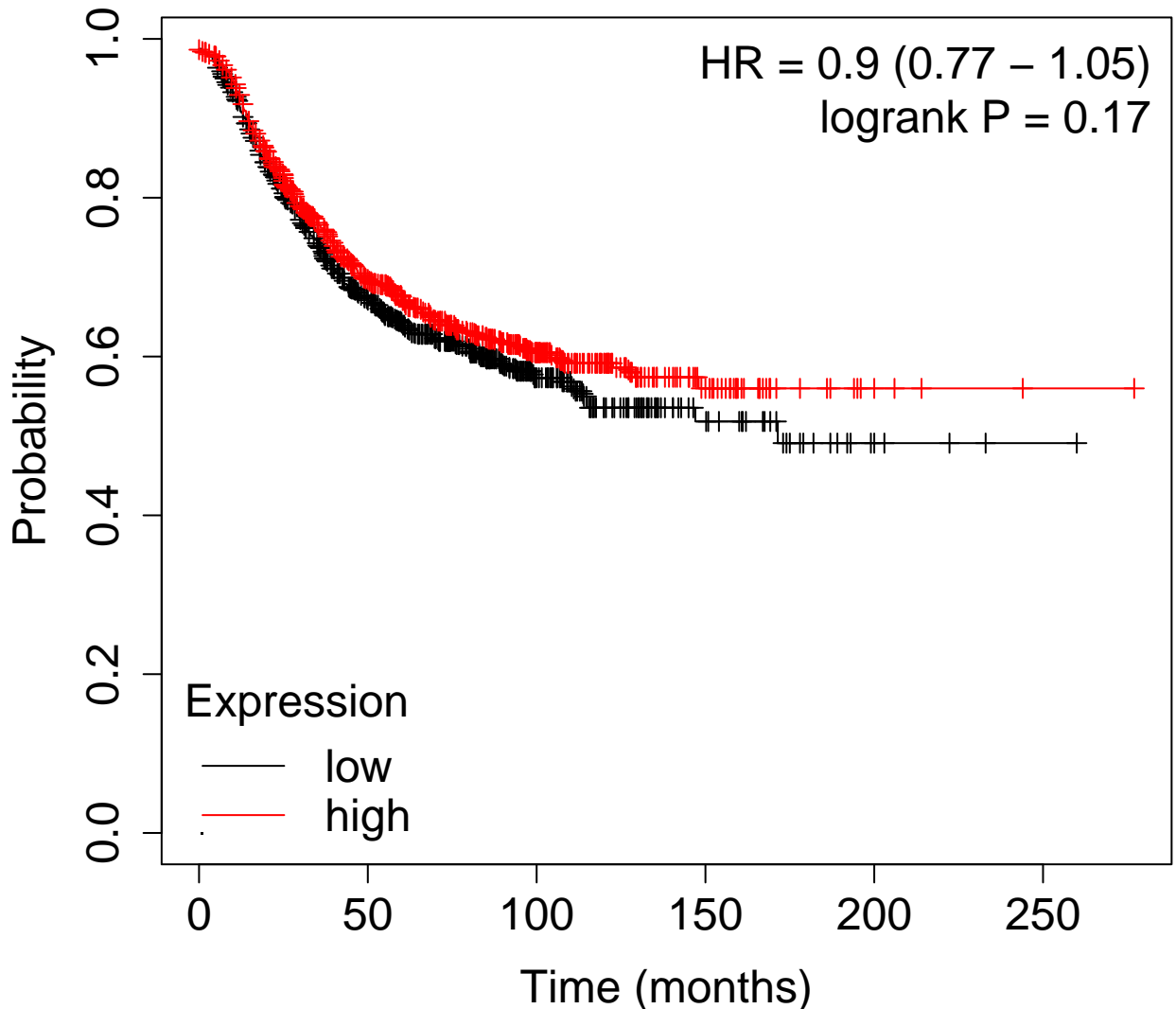

Number at risk

|      |     |     |     |    |   |   |
|------|-----|-----|-----|----|---|---|
| low  | 882 | 493 | 134 | 30 | 5 | 1 |
| high | 882 | 484 | 211 | 38 | 5 | 1 |

Supplement: Figure 6—source data 1. [file elife-65418-fig6-data1.zip › Fig 6/C/KM plotter/SLC35F5.pdf]

# ZNF710 (239700\_at)

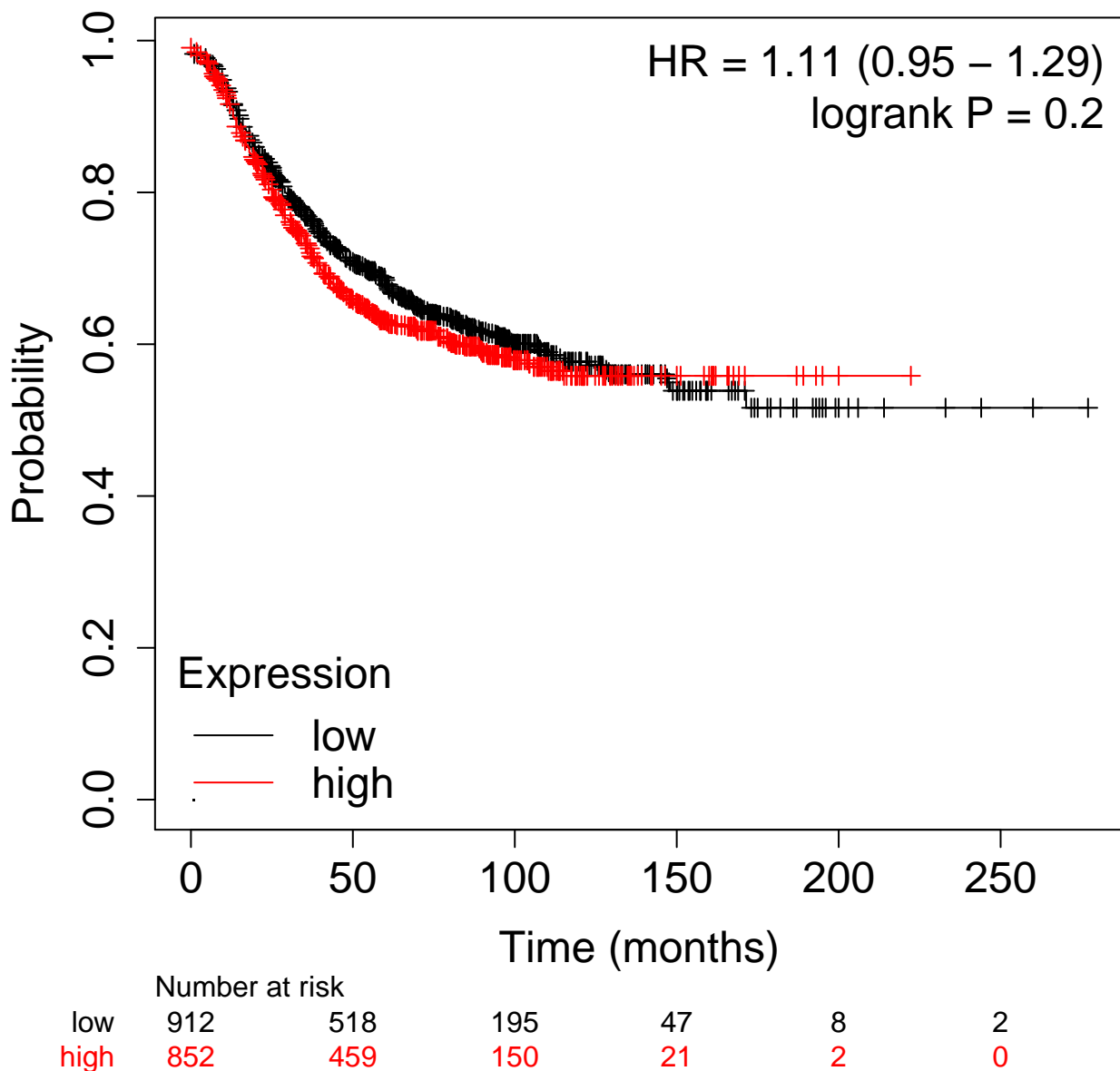

Supplement: Figure 6—source data 1. [file elife-65418-fig6-data1.zip › Fig 6/C/KM plotter/ZNF710.pdf]

# RHBDL2 (1552502\_s\_at)

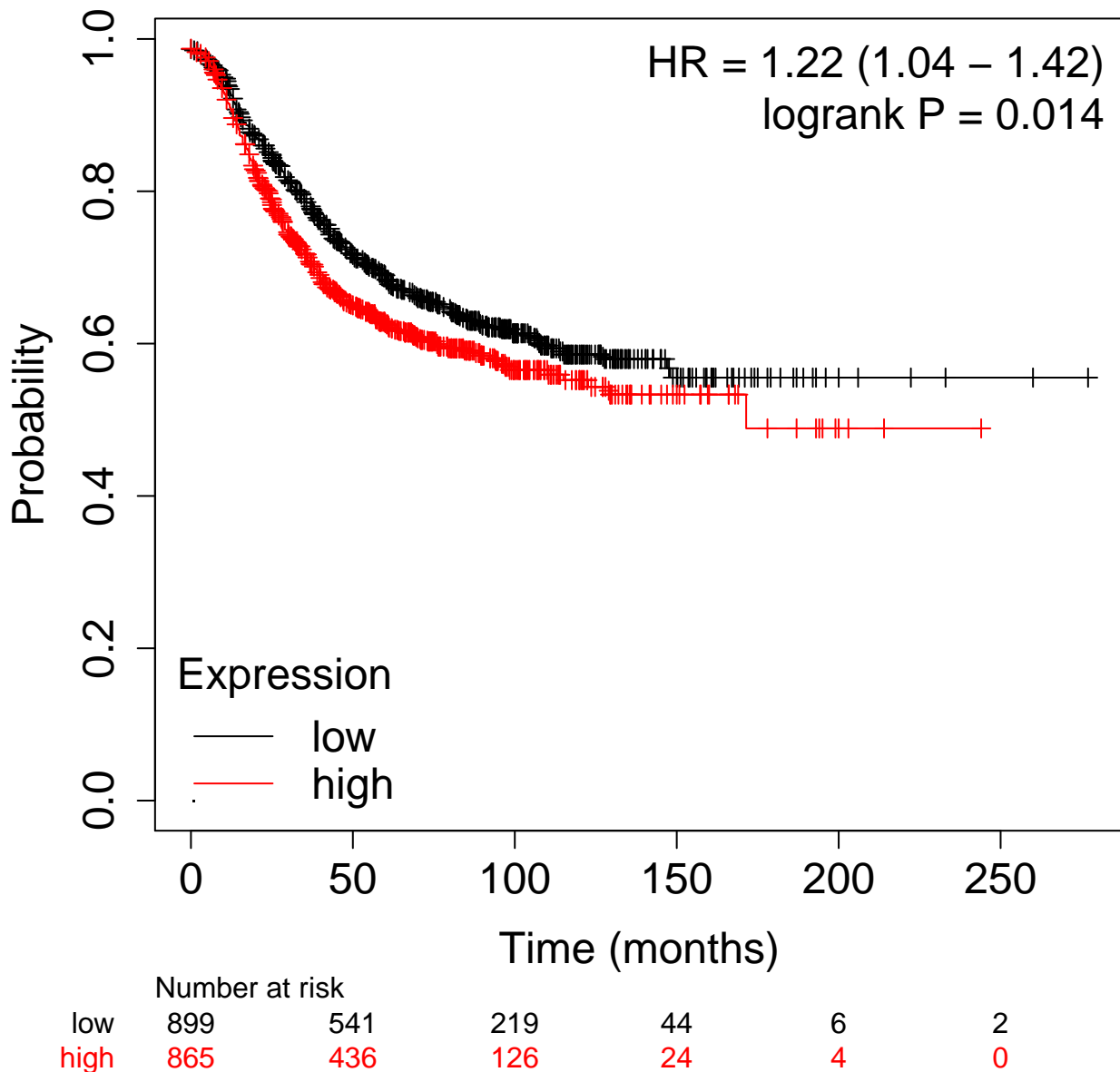

Supplement: Figure 6—source data 1. [file elife-65418-fig6-data1.zip › Fig 6/C/KM plotter/RHBDL2.pdf]

# PPME1 (217841\_s\_at)

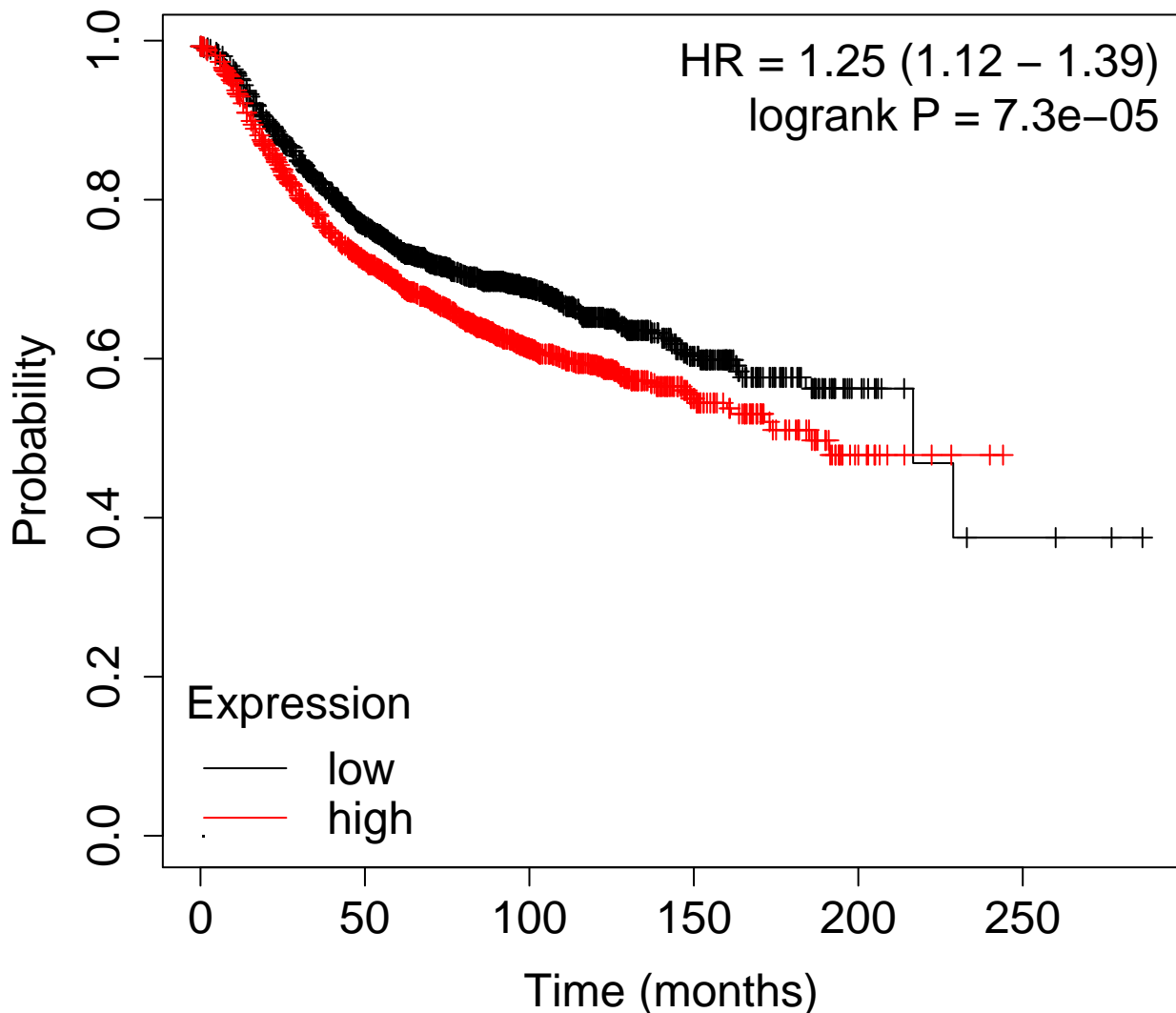

Number at risk

|      |      |      |     |     |    |   |
|------|------|------|-----|-----|----|---|
| low  | 1976 | 1294 | 571 | 139 | 14 | 3 |
| high | 1975 | 1225 | 504 | 102 | 13 | 0 |

Supplement: Figure 6—source data 1. [file elife-65418-fig6-data1.zip › Fig 6/C/KM plotter/PPME1.pdf]

# ARL6IP1 (211935\_at)

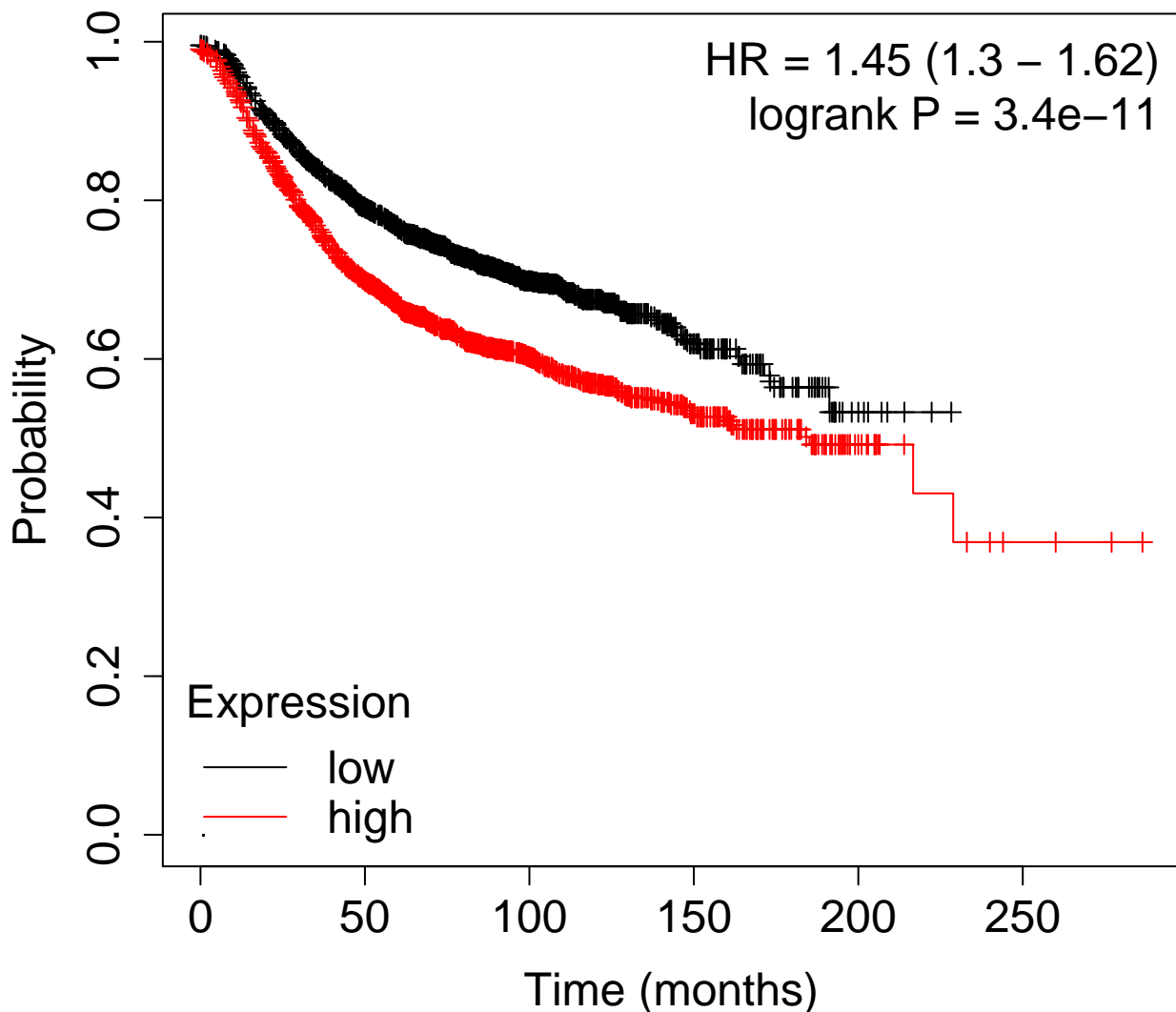

Number at risk

|      |      |      |     |     |    |   |
|------|------|------|-----|-----|----|---|
| low  | 1976 | 1350 | 572 | 105 | 8  | 0 |
| high | 1975 | 1169 | 503 | 136 | 19 | 3 |

Supplement: Figure 6—source data 1. [file elife-65418-fig6-data1.zip › Fig 6/C/KM plotter/ARL6IP1.pdf]

# MED22 (206593\_s\_at)

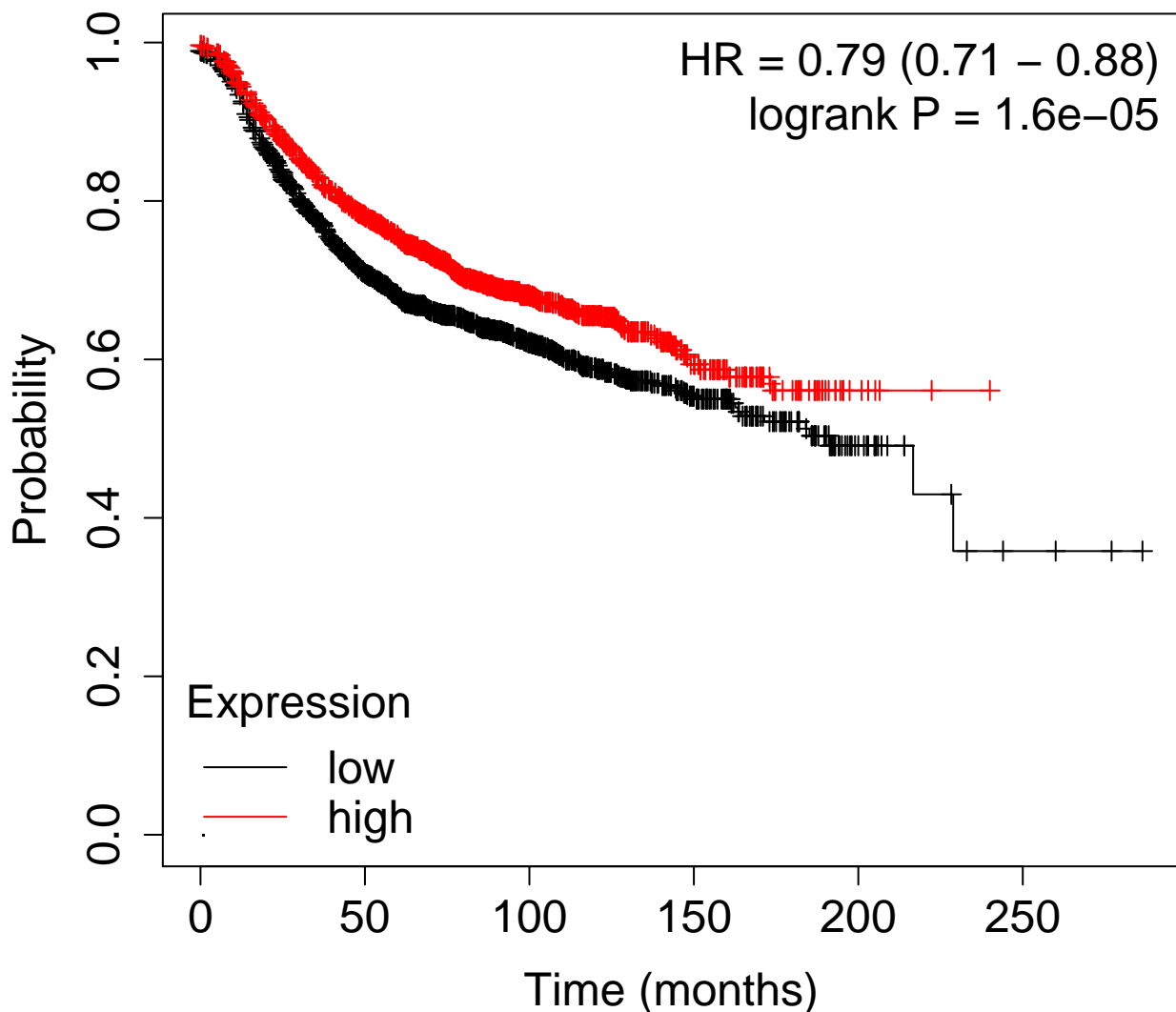

Number at risk

|      |      |      |     |     |    |   |
|------|------|------|-----|-----|----|---|
| low  | 1981 | 1178 | 518 | 148 | 21 | 3 |
| high | 1970 | 1341 | 557 | 93  | 6  | 0 |

Supplement: Figure 6—source data 1. [file elife-65418-fig6-data1.zip › Fig 6/C/KM plotter/MED22.pdf]

# DSCC1 (219000\_s\_at)

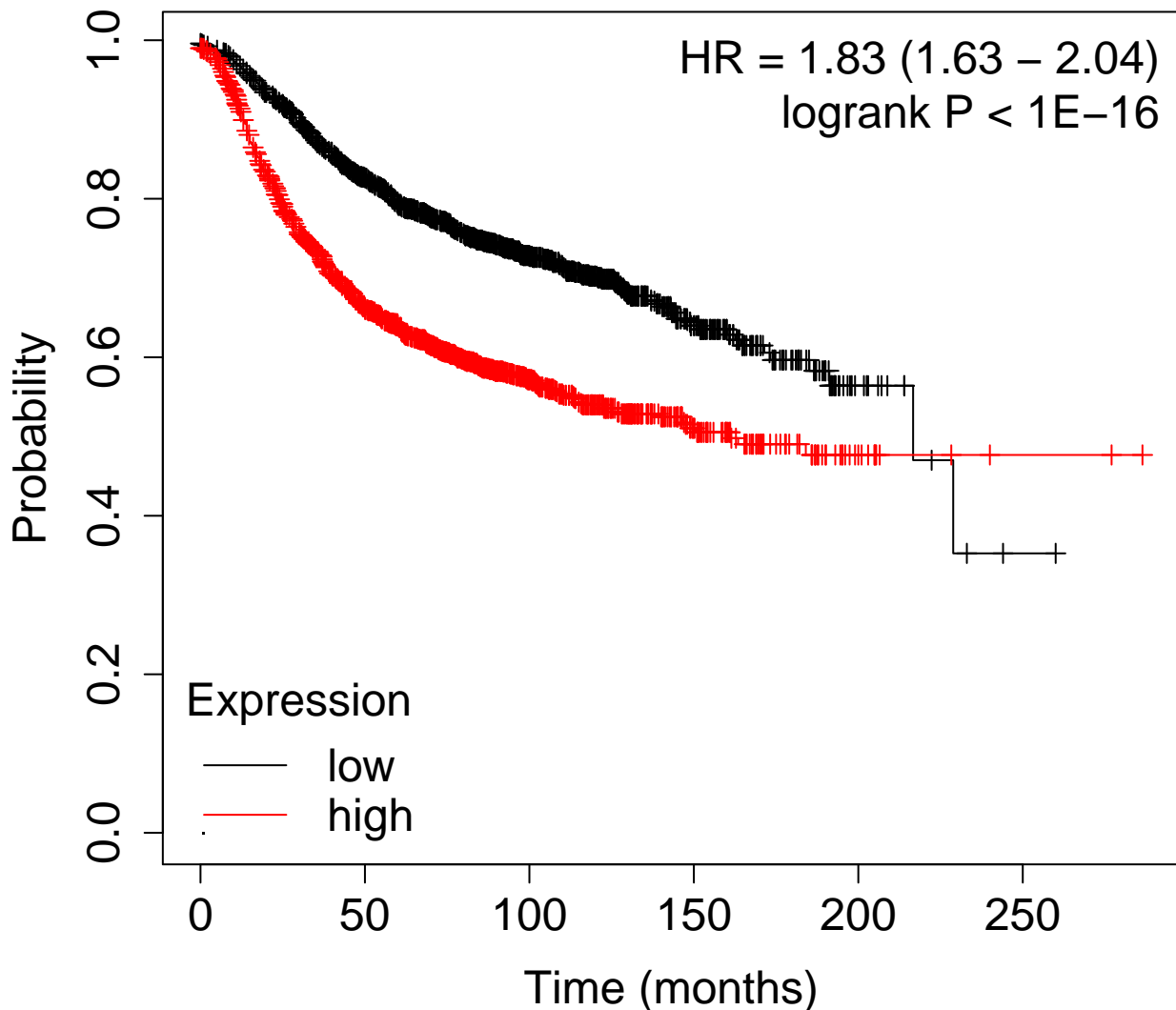

Number at risk

|      |      |      |     |     |    |   |
|------|------|------|-----|-----|----|---|
| low  | 1981 | 1424 | 628 | 141 | 14 | 1 |
| high | 1970 | 1095 | 447 | 100 | 13 | 2 |

Supplement: Figure 6—source data 1. [file elife-65418-fig6-data1.zip › Fig 6/C/KM plotter/DSCC1.pdf]

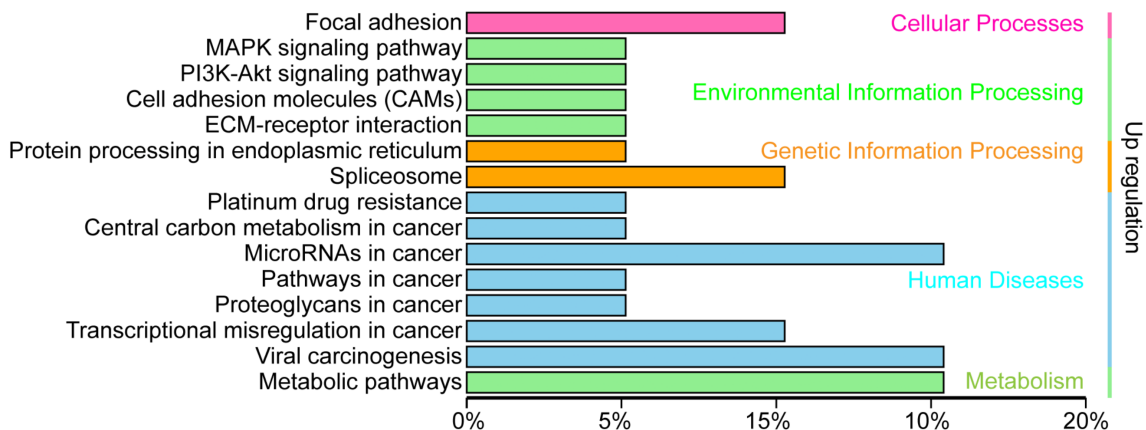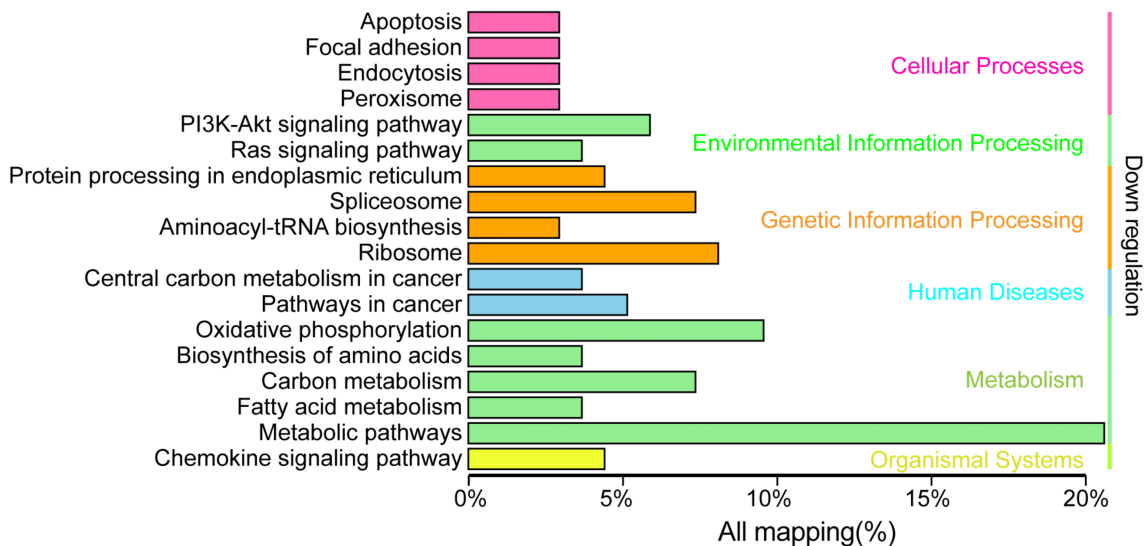

Supplement: Figure 6—figure supplement 1—source data 1. [file elife-65418-fig6-figsupp1-data1.zip › Fig 6-Supplement fig 1/KEGG.pdf]

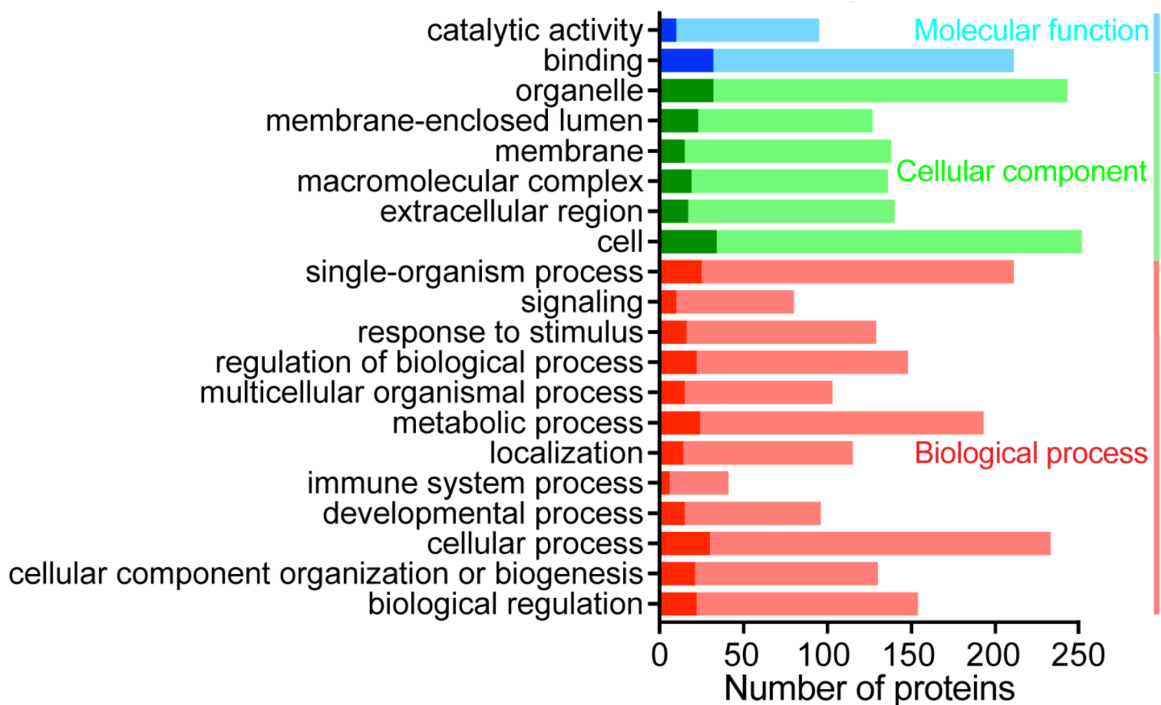

Supplement: Figure 6—figure supplement 1—source data 1. [file elife-65418-fig6-figsupp1-data1.zip › Fig 6-Supplement fig 1/GO.pdf]

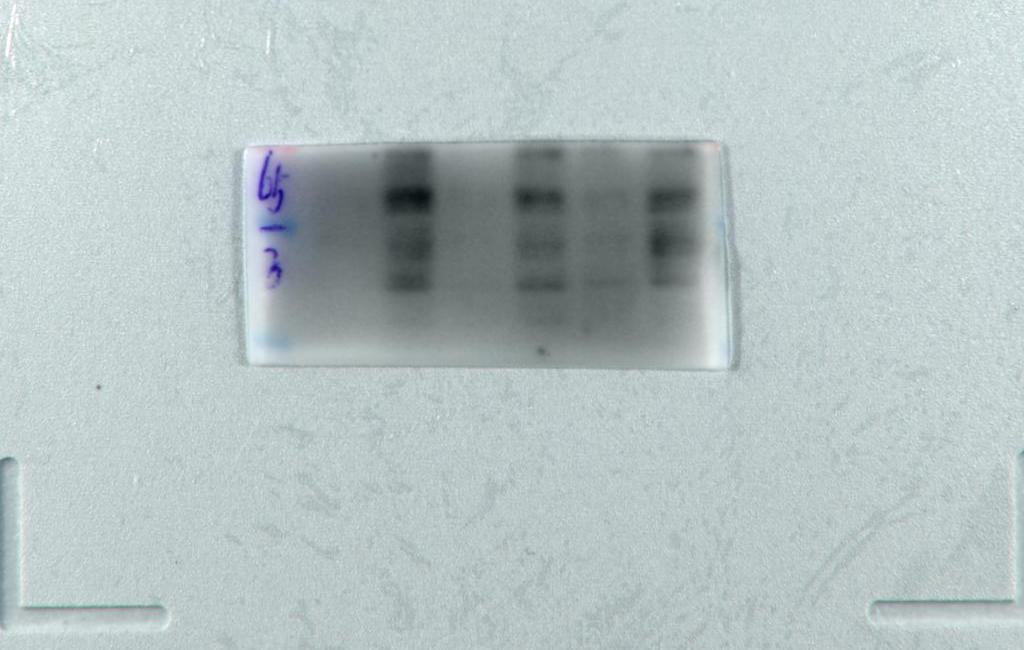

Supplement: Figure 7—source data 1. [file elife-65418-fig7-data1.zip › elife-65418-fig7-data1-v3/A/468 p-YAP1.jpg]

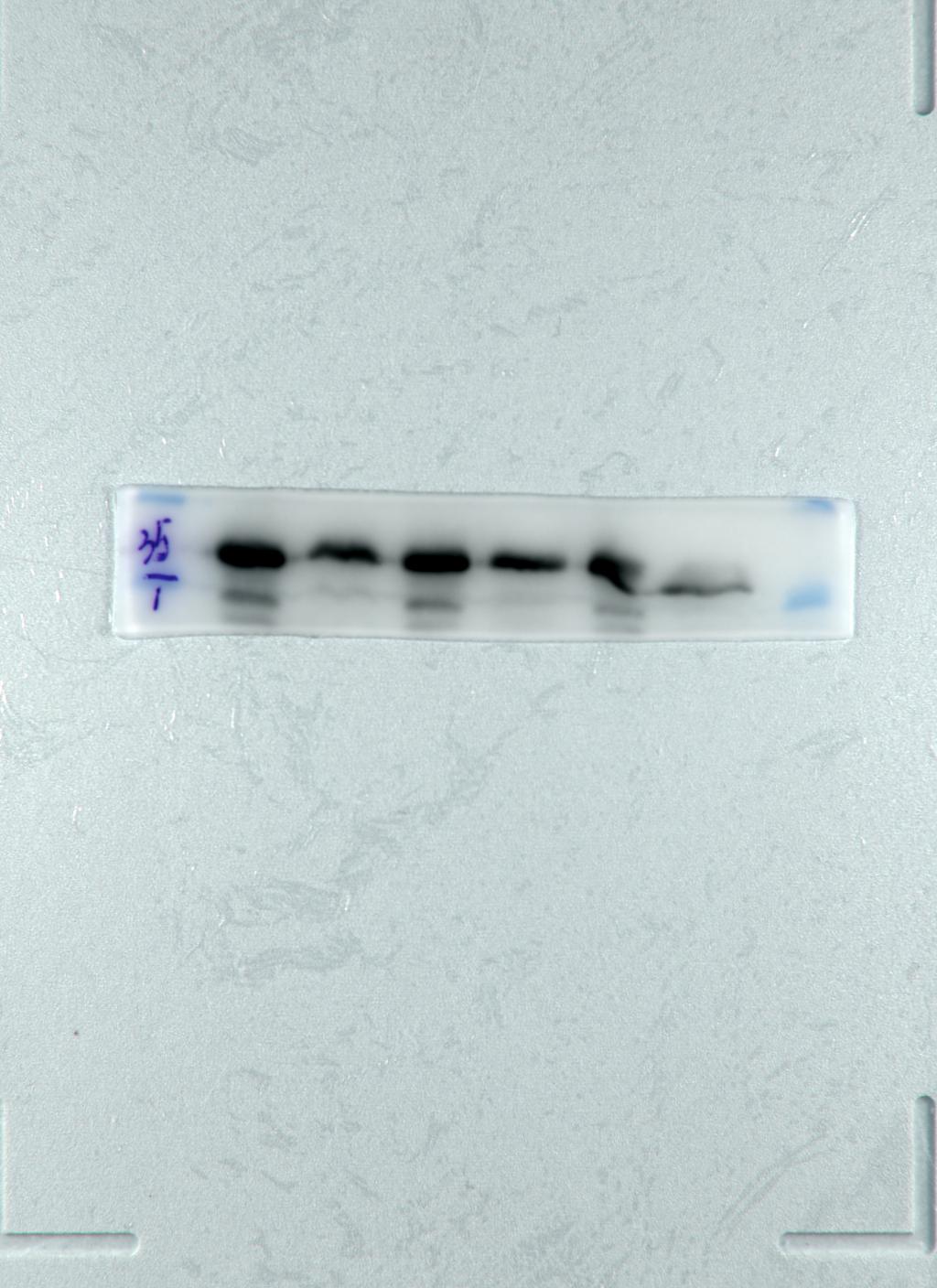

Supplement: Figure 7—source data 1. [file elife-65418-fig7-data1.zip › elife-65418-fig7-data1-v3/A/231 RHBDL2.jpg]

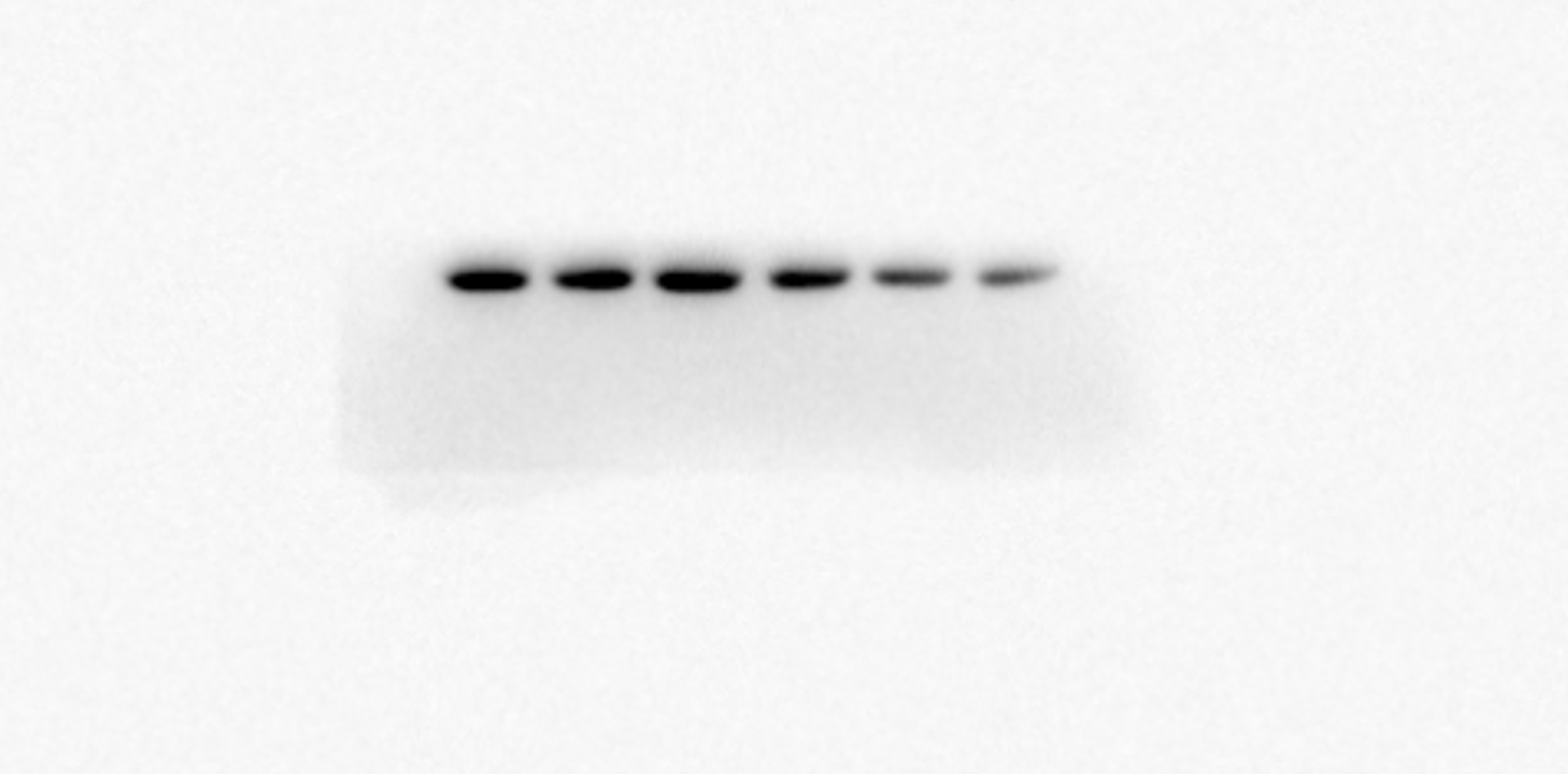

Supplement: Figure 7—source data 1. [file elife-65418-fig7-data1.zip › elife-65418-fig7-data1-v3/A/468 GAPDH.tif]

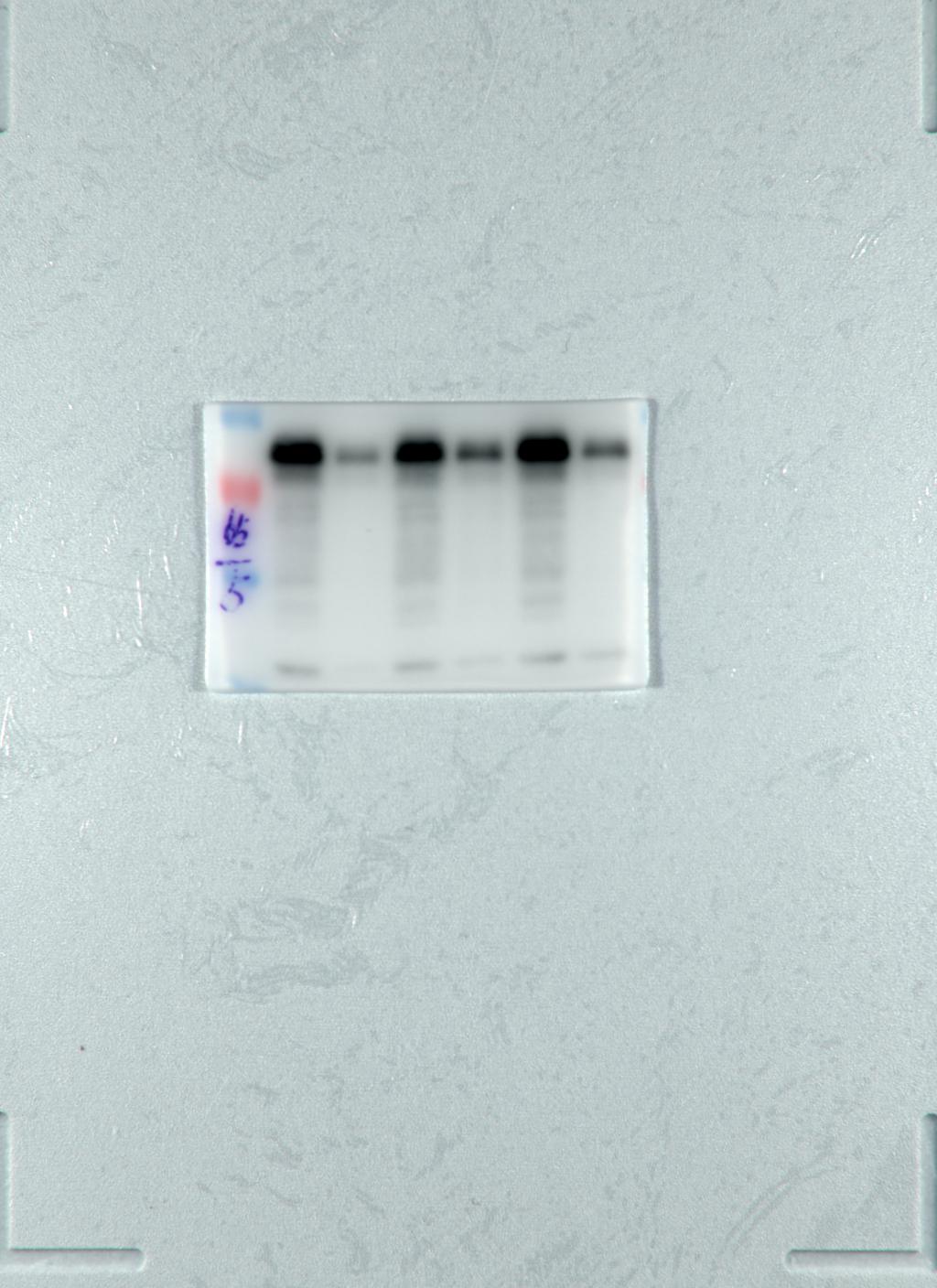

Supplement: Figure 7—source data 1. [file elife-65418-fig7-data1.zip › elife-65418-fig7-data1-v3/A/468 YAP1.jpg]

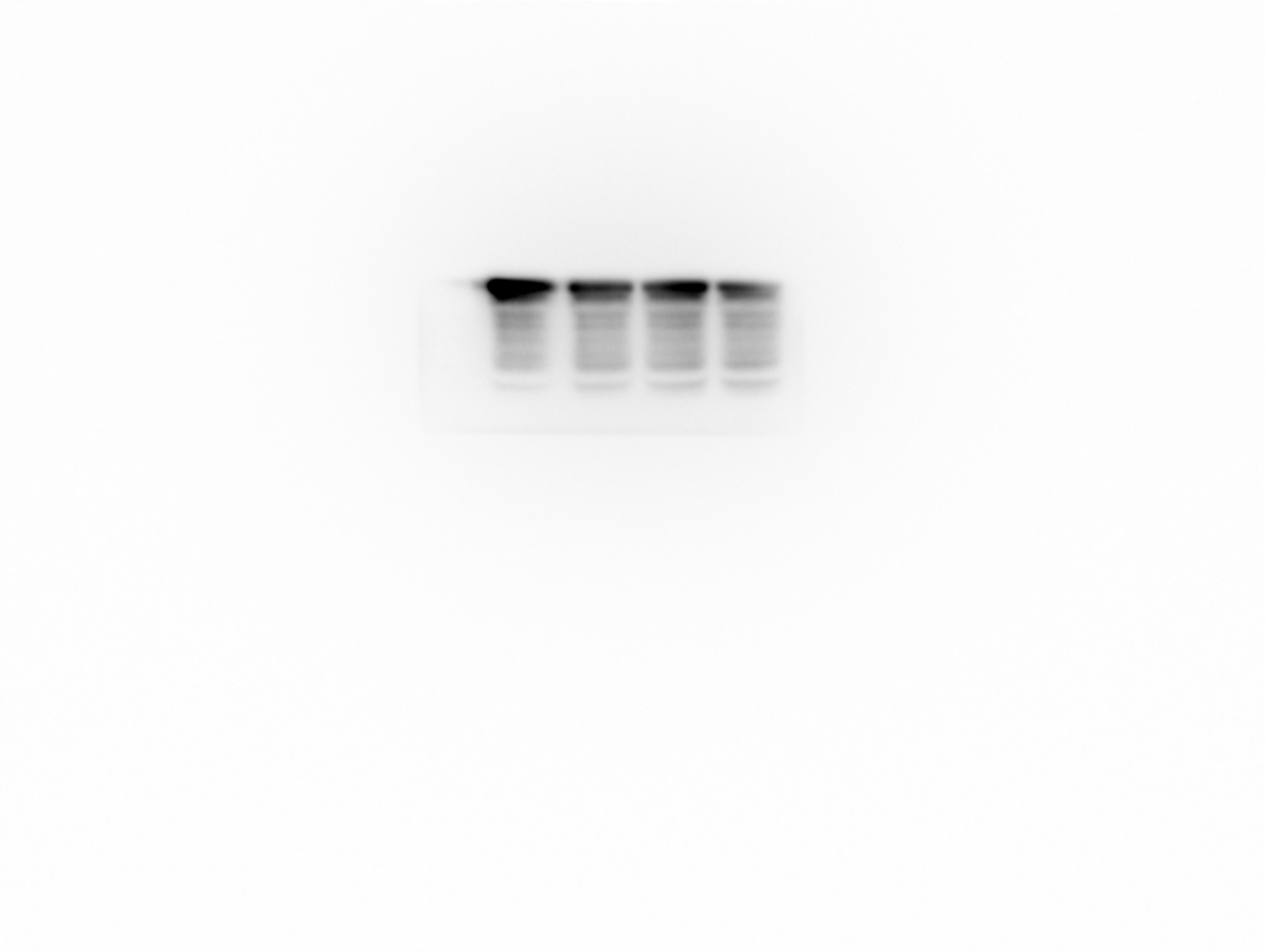

Supplement: Figure 7—source data 1. [file elife-65418-fig7-data1.zip › elife-65418-fig7-data1-v3/A/231 YAP1.tif]

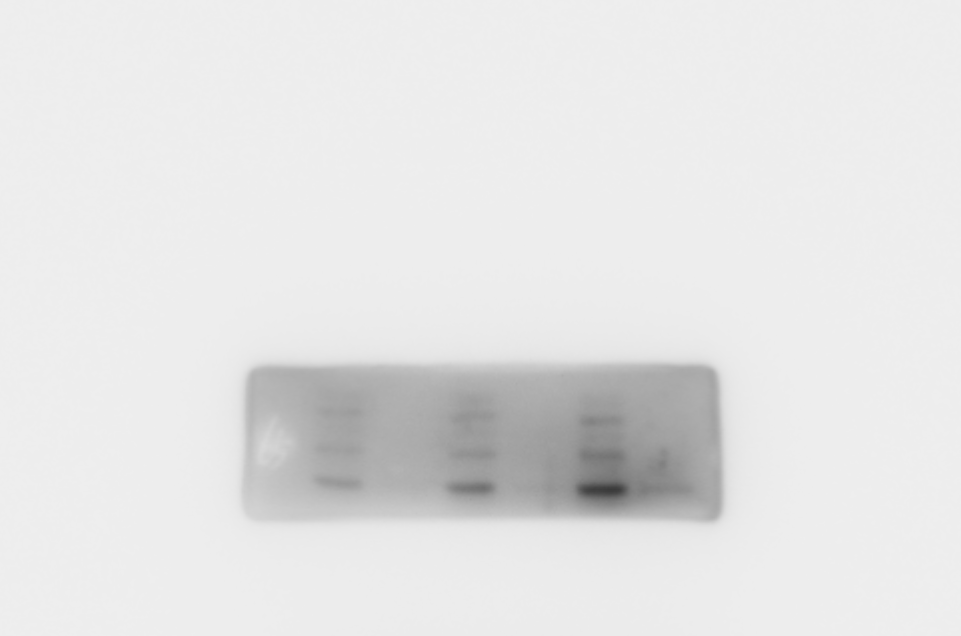

Supplement: Figure 7—source data 1. [file elife-65418-fig7-data1.zip › elife-65418-fig7-data1-v3/A/468 RHBDL2.tif]

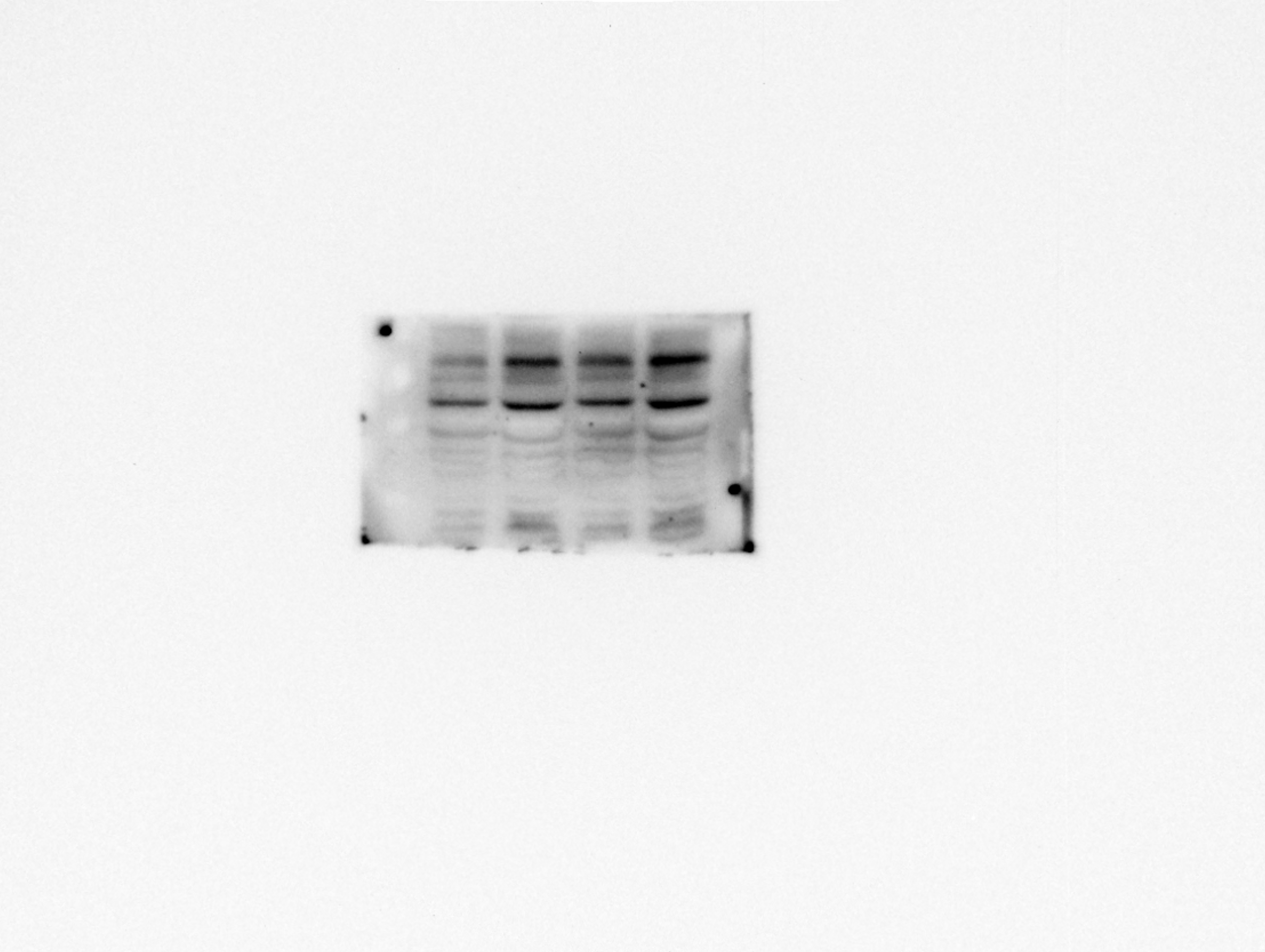

Supplement: Figure 7—source data 1. [file elife-65418-fig7-data1.zip › elife-65418-fig7-data1-v3/A/231 p yap.tif]

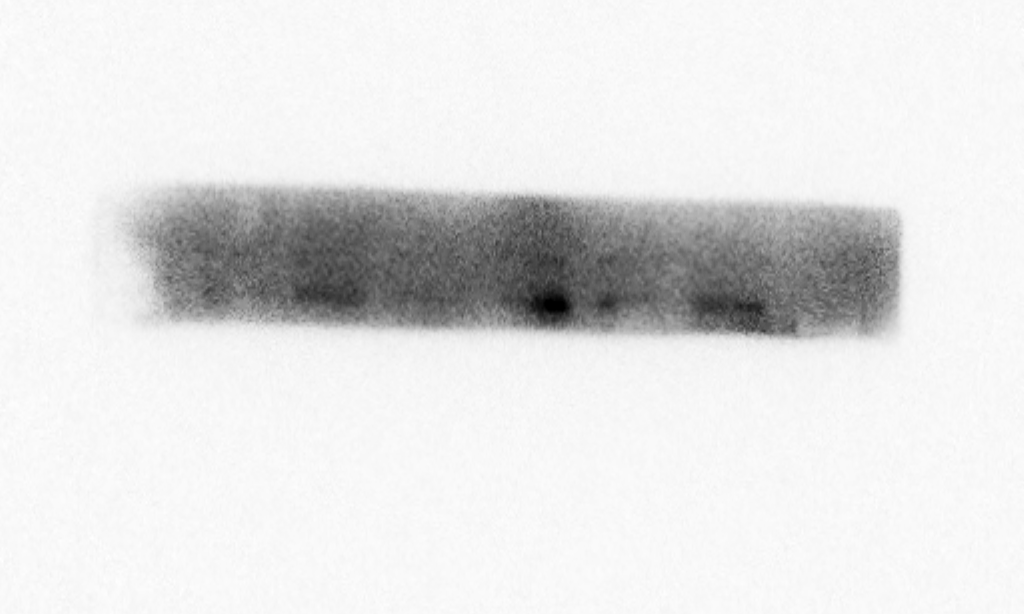

Supplement: Figure 7—source data 1. [file elife-65418-fig7-data1.zip › elife-65418-fig7-data1-v3/C/8. 468 Actin Nucleus.tif]

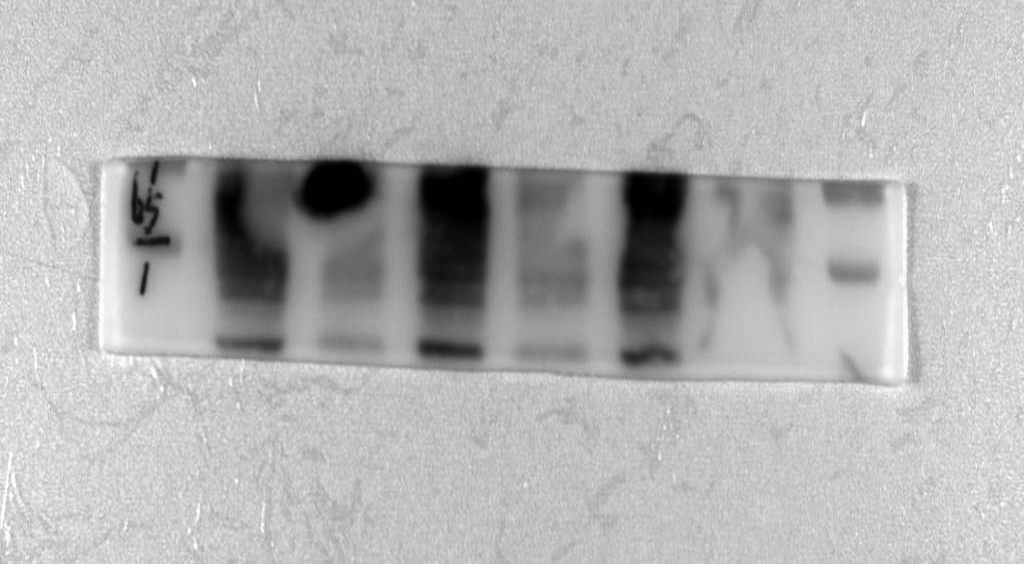

Supplement: Figure 7—source data 1. [file elife-65418-fig7-data1.zip › elife-65418-fig7-data1-v3/C/5. 468 YAP1 Cytoplasm.jpg]

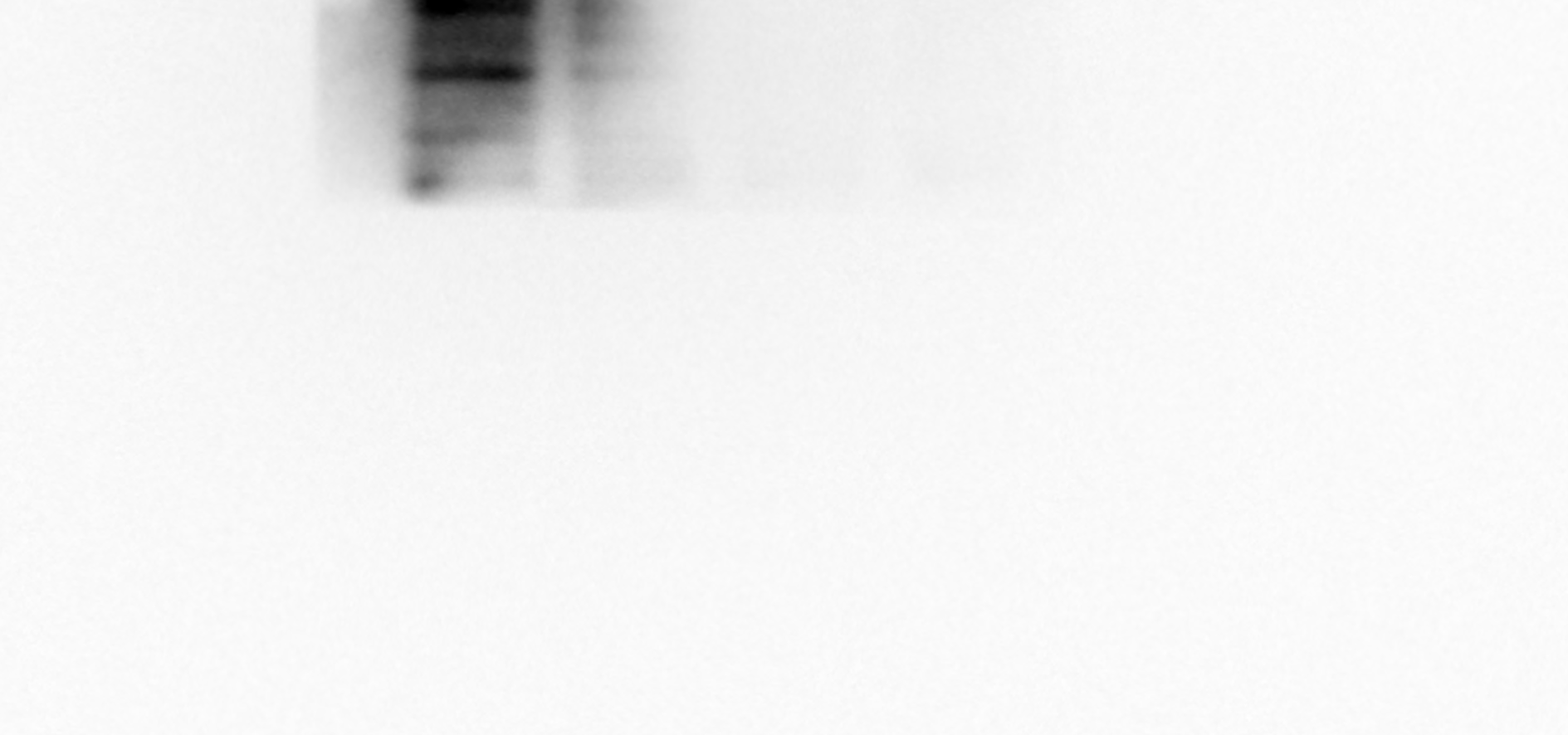

Supplement: Figure 7—source data 1. [file elife-65418-fig7-data1.zip › elife-65418-fig7-data1-v3/C/2. 231 YAP1 Nucleus.tif]

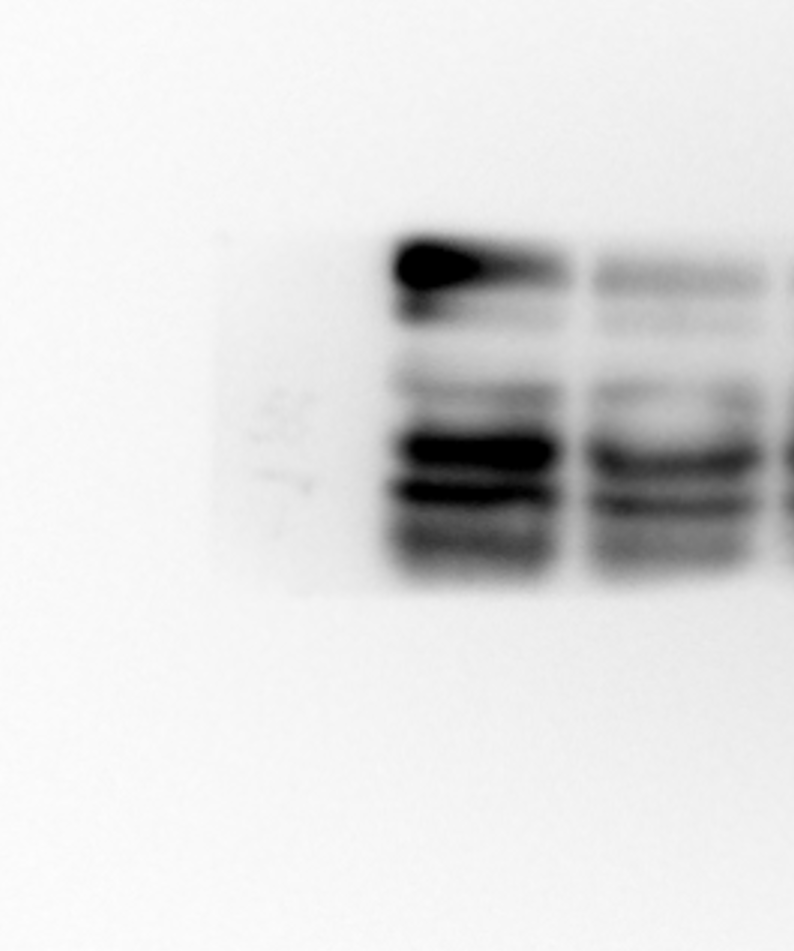

Supplement: Figure 7—source data 1. [file elife-65418-fig7-data1.zip › elife-65418-fig7-data1-v3/C/1. 231 YAP1 Cytoplasm.tif]

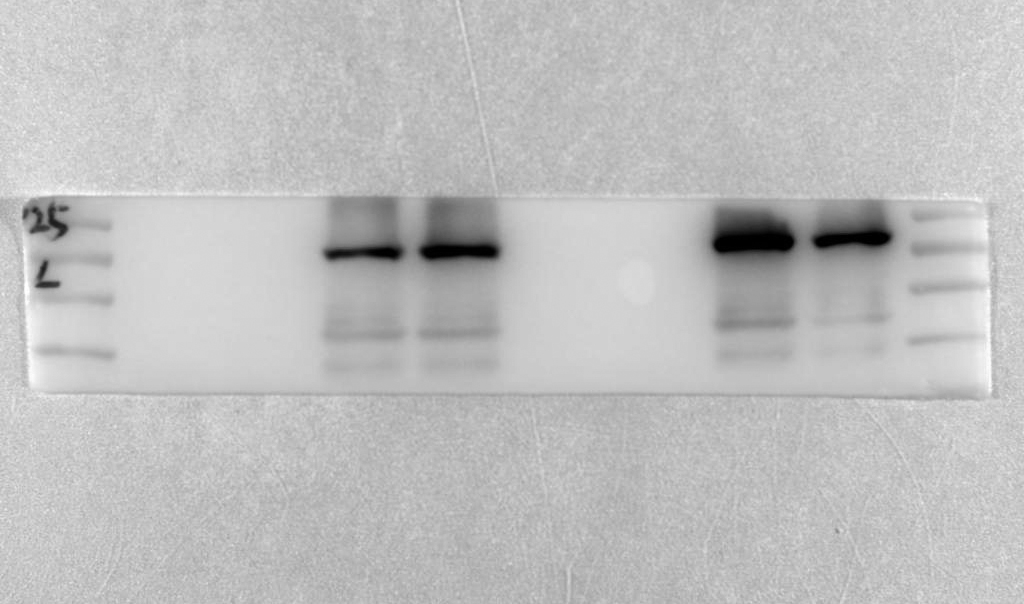

Supplement: Figure 7—source data 1. [file elife-65418-fig7-data1.zip › elife-65418-fig7-data1-v3/C/9. 468 Lamin Cytoplasm+Nucleus.jpg]

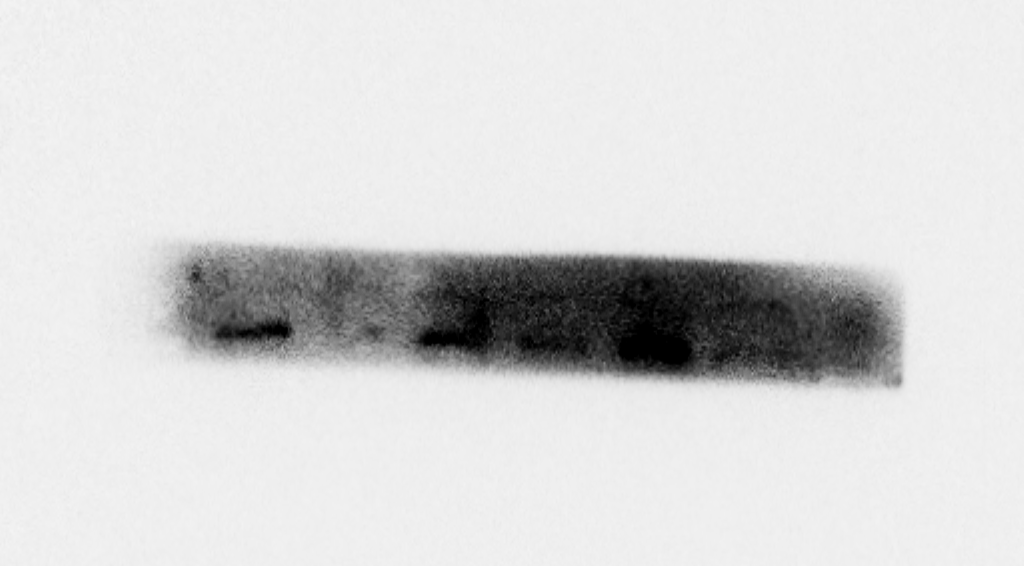

Supplement: Figure 7—source data 1. [file elife-65418-fig7-data1.zip › elife-65418-fig7-data1-v3/C/6. 468 YAP1 Nucleus.tif]

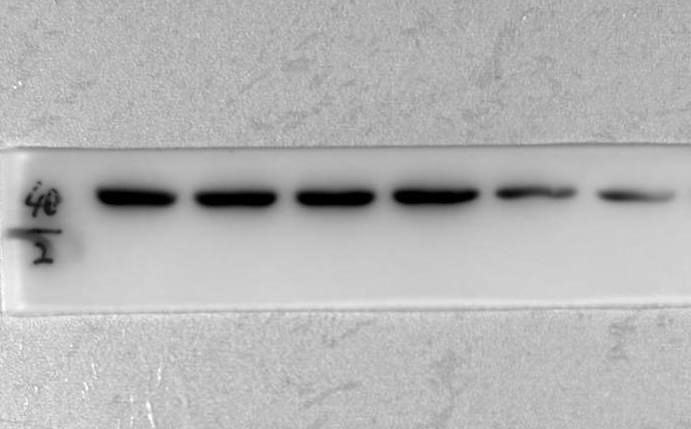

Supplement: Figure 7—source data 1. [file elife-65418-fig7-data1.zip › elife-65418-fig7-data1-v3/C/7. 468 Actin Cytoplasm.jpg]

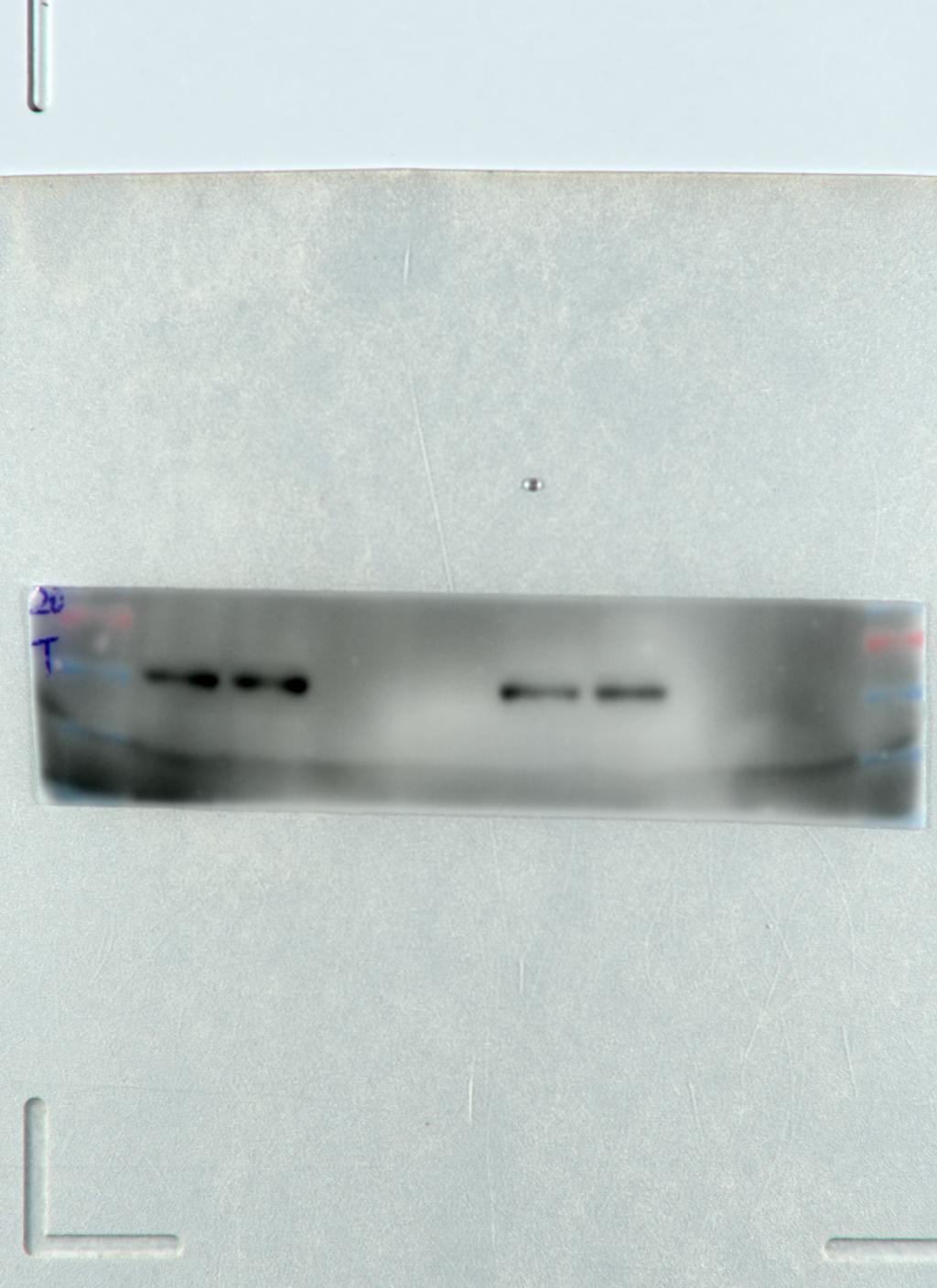

Supplement: Figure 7—source data 1. [file elife-65418-fig7-data1.zip › elife-65418-fig7-data1-v3/C/3 231 Tubulin Cytoplasm+Nucleus.jpg]

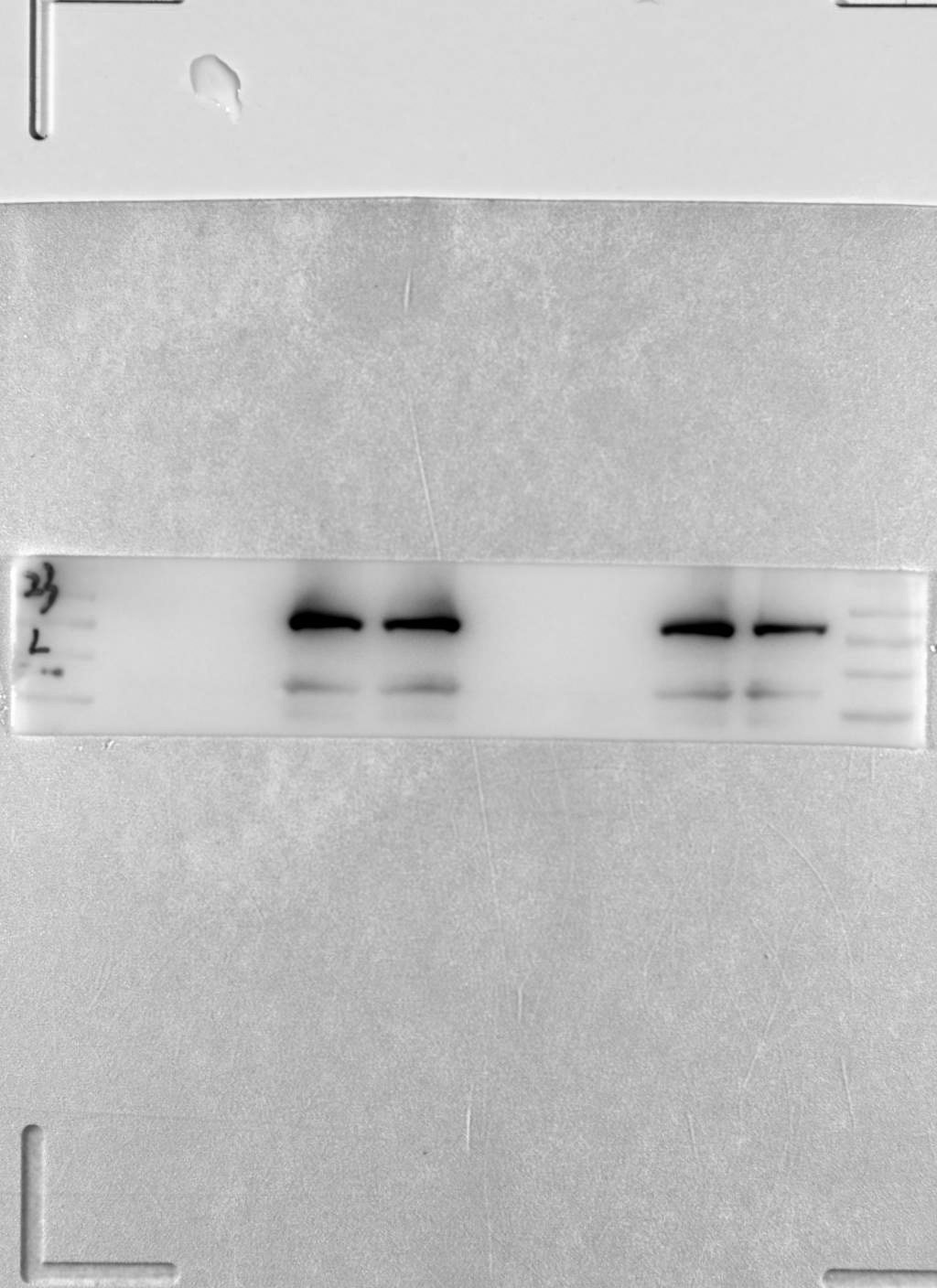

Supplement: Figure 7—source data 1. [file elife-65418-fig7-data1.zip › elife-65418-fig7-data1-v3/C/4. 231 Lamin Cytoplasm+Nucleus.jpg]

# BD FACSDiva 8.0.1

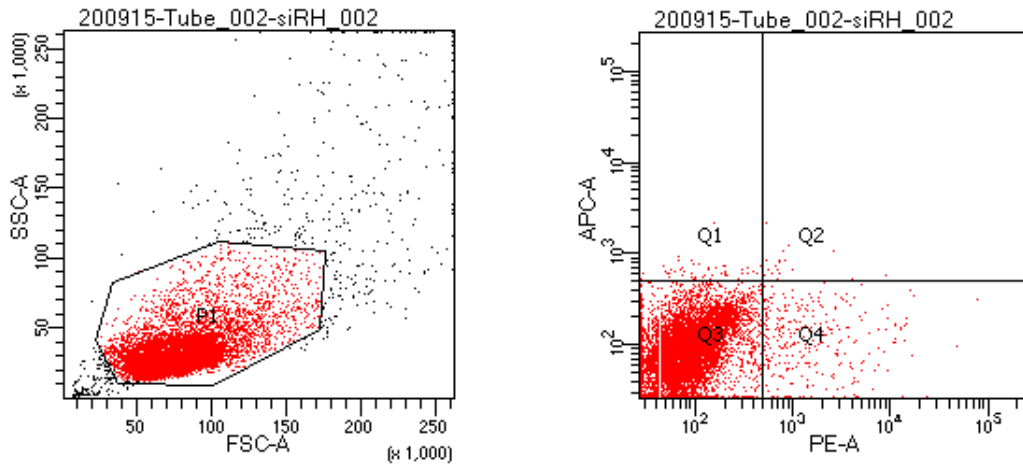

| Tube: Tube_002-siRH_002 |         |         |        |
|-------------------------|---------|---------|--------|
| Population              | #Events | %Parent | %Total |
| ■ All Events            | 10,000  | ####    | 100.0  |
| ■ P1                    | 9,418   | 94.2    | 94.2   |
| ☒ Q1                    | 49      | 0.5     | 0.5    |
| ☒ Q2                    | 18      | 0.2     | 0.2    |
| ☒ Q3                    | 9,060   | 96.2    | 90.6   |
| ☒ Q4                    | 291     | 3.1     | 2.9    |

Supplement: Figure 7—source data 1. [file elife-65418-fig7-data1.zip › elife-65418-fig7-data1-v3/D/siRH.pdf]

# BD FACSDiva 8.0.1

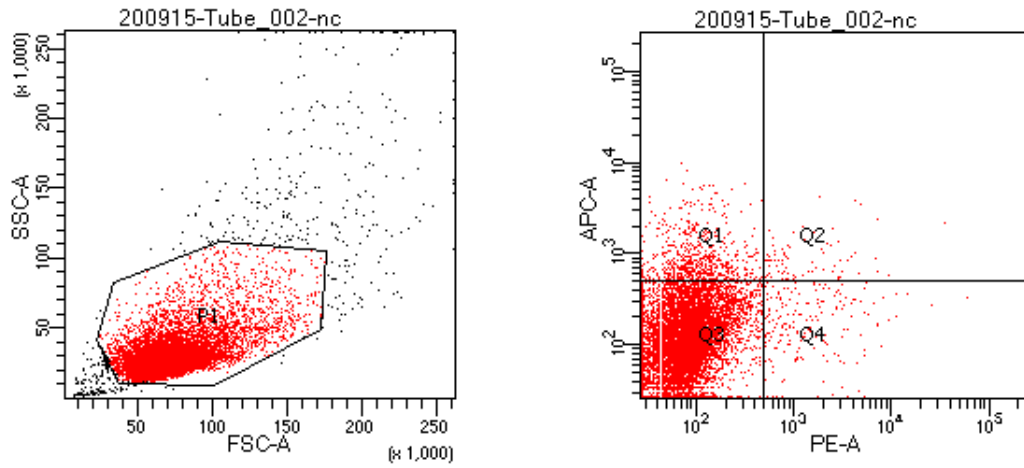

| Tube: Tube_002-nc |         |         |        |
|-------------------|---------|---------|--------|
| Population        | #Events | %Parent | %Total |
| ■ All Events      | 10,000  | ####    | 100.0  |
| ■ P1              | 9,475   | 94.8    | 94.8   |
| ☒ Q1              | 613     | 6.5     | 6.1    |
| ☒ Q2              | 78      | 0.8     | 0.8    |
| ☒ Q3              | 8,592   | 90.7    | 85.9   |
| ☒ Q4              | 192     | 2.0     | 1.9    |

Supplement: Figure 7—source data 1. [file elife-65418-fig7-data1.zip › elife-65418-fig7-data1-v3/D/NC.pdf]

# BD FACSDiva 8.0.1

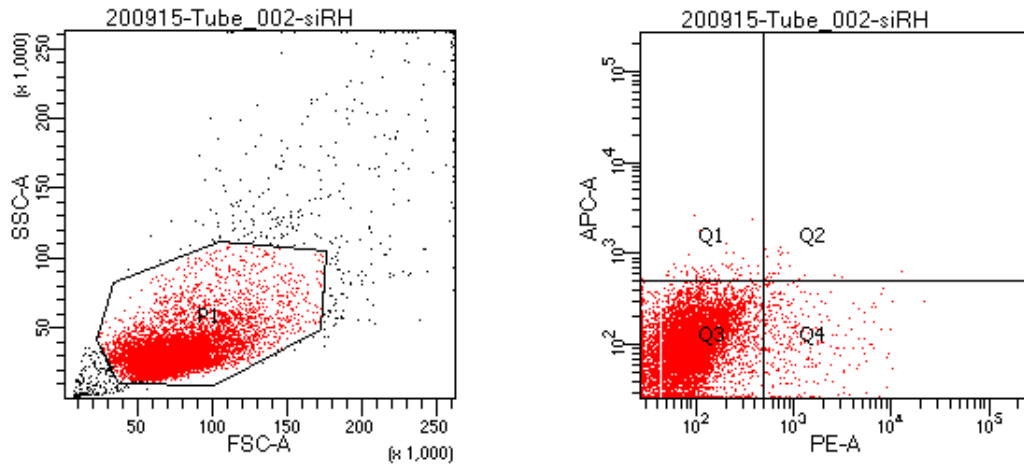

| Tube: Tube_002-siRH |         |         |        |
|---------------------|---------|---------|--------|
| Population          | #Events | %Parent | %Total |
| ■ All Events        | 10,000  | ####    | 100.0  |
| ■ P1                | 9,425   | 94.2    | 94.2   |
| ☒ Q1                | 69      | 0.7     | 0.7    |
| ☒ Q2                | 23      | 0.2     | 0.2    |
| ☒ Q3                | 9,045   | 96.0    | 90.4   |
| ☒ Q4                | 288     | 3.1     | 2.9    |

Supplement: Figure 7—source data 1. [file elife-65418-fig7-data1.zip › elife-65418-fig7-data1-v3/E/468 RH.pdf]

# BD FACSDiva 8.0.1

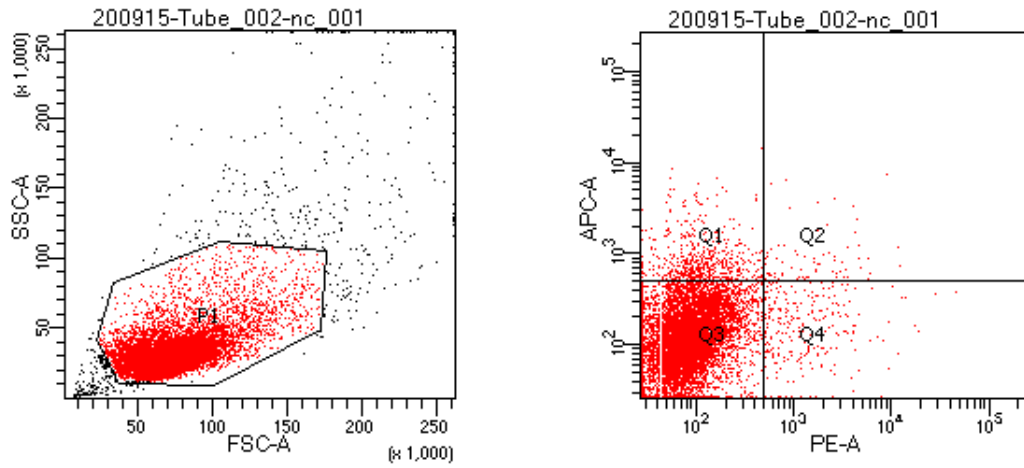

| Tube: Tube_002-nc_001 |         |         |        |
|-----------------------|---------|---------|--------|
| Population            | #Events | %Parent | %Total |
| ■ All Events          | 10,000  | ####    | 100.0  |
| ■ P1                  | 9,397   | 94.0    | 94.0   |
| ☒ Q1                  | 520     | 5.5     | 5.2    |
| ☒ Q2                  | 72      | 0.8     | 0.7    |
| ☒ Q3                  | 8,553   | 91.0    | 85.5   |
| ☒ Q4                  | 252     | 2.7     | 2.5    |

Supplement: Figure 7—source data 1. [file elife-65418-fig7-data1.zip › elife-65418-fig7-data1-v3/E/468 NC.pdf]

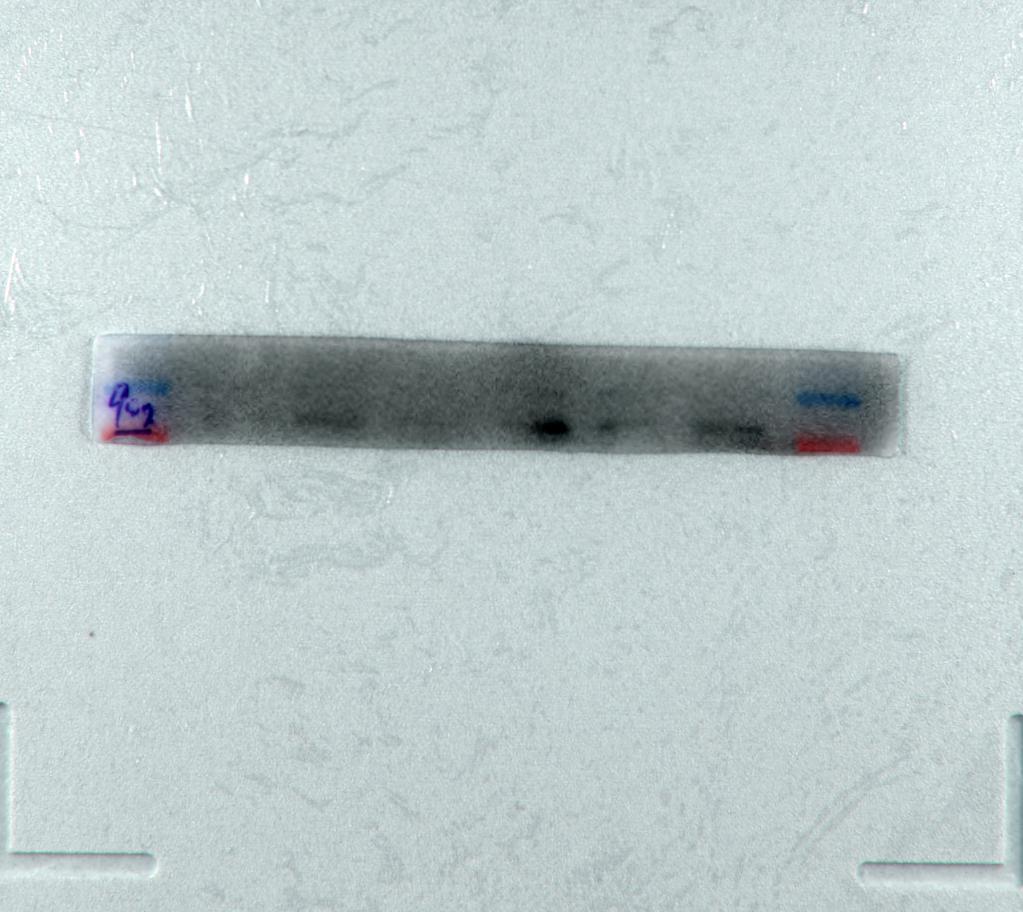

Supplement: Figure 7—source data 1. [file elife-65418-fig7-data1.zip › elife-65418-fig7-data1-v3/B/468 USP31.jpg]

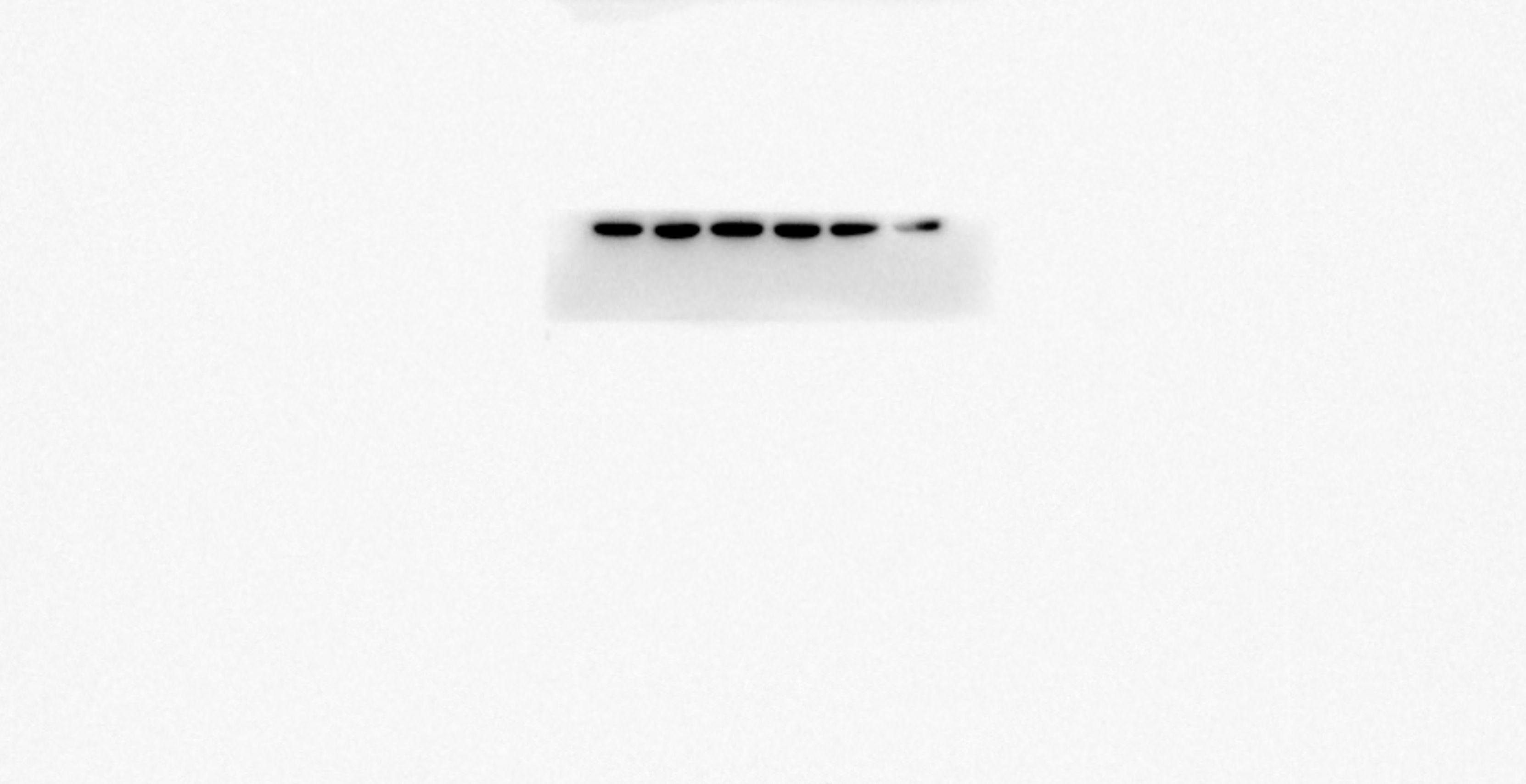

Supplement: Figure 7—source data 1. [file elife-65418-fig7-data1.zip › elife-65418-fig7-data1-v3/B/468 GAPDH.tif]

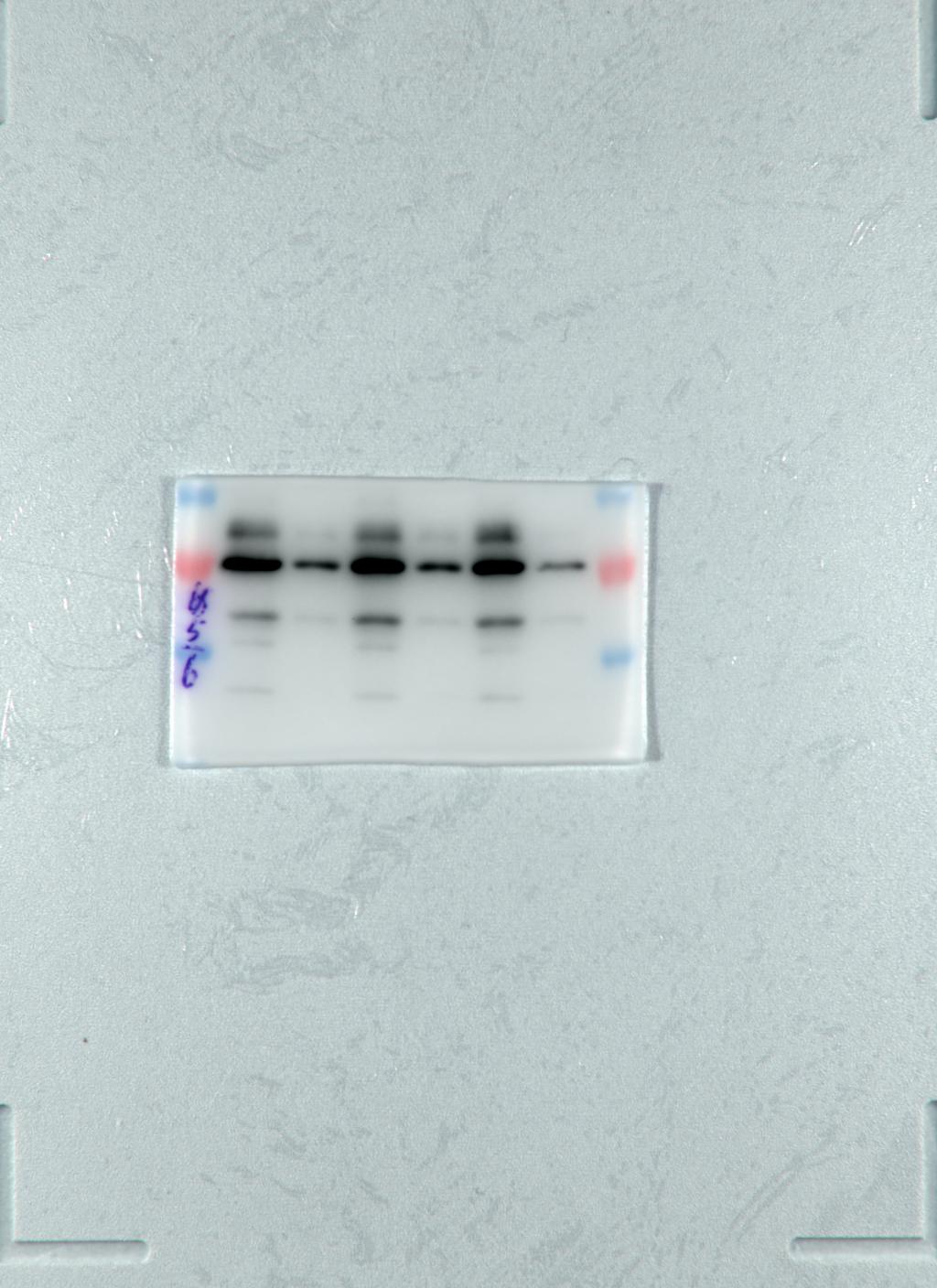

Supplement: Figure 7—source data 1. [file elife-65418-fig7-data1.zip › elife-65418-fig7-data1-v3/B/468 yap.jpg]

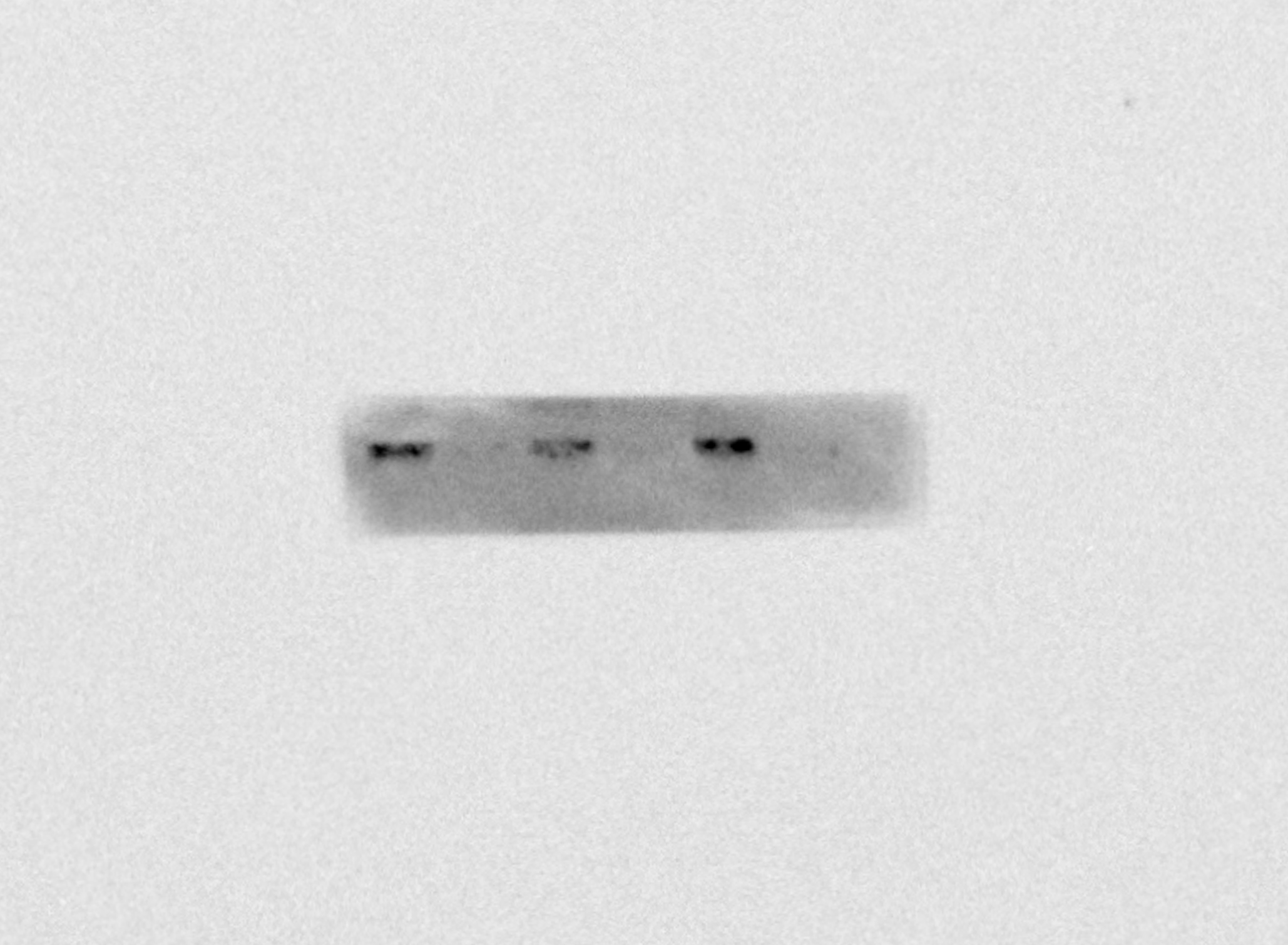

Supplement: Figure 7—source data 1. [file elife-65418-fig7-data1.zip › elife-65418-fig7-data1-v3/B/468 RHBDL2.tif]

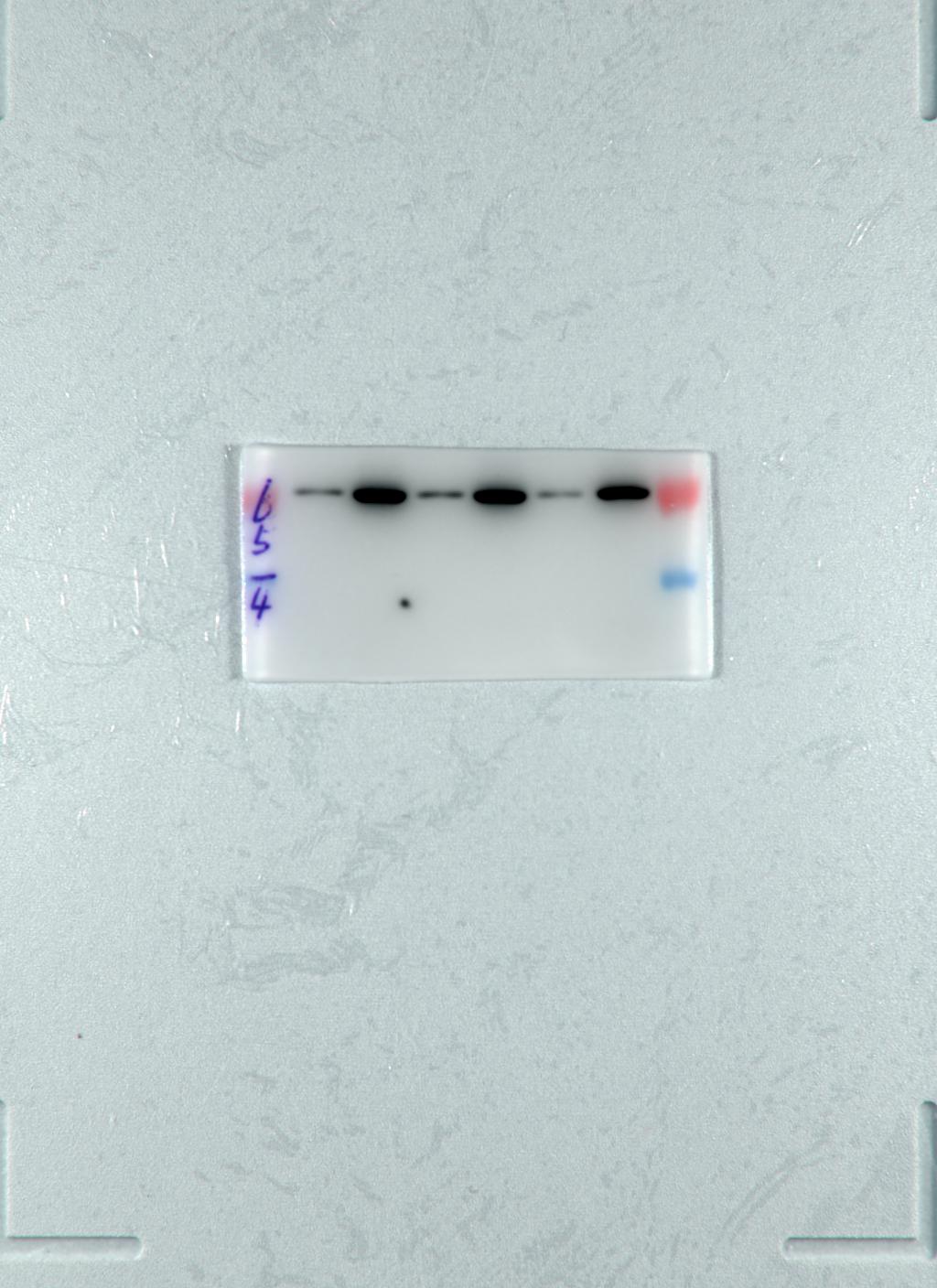

Supplement: Figure 7—source data 1. [file elife-65418-fig7-data1.zip › elife-65418-fig7-data1-v3/B/468 p-YAP.jpg]

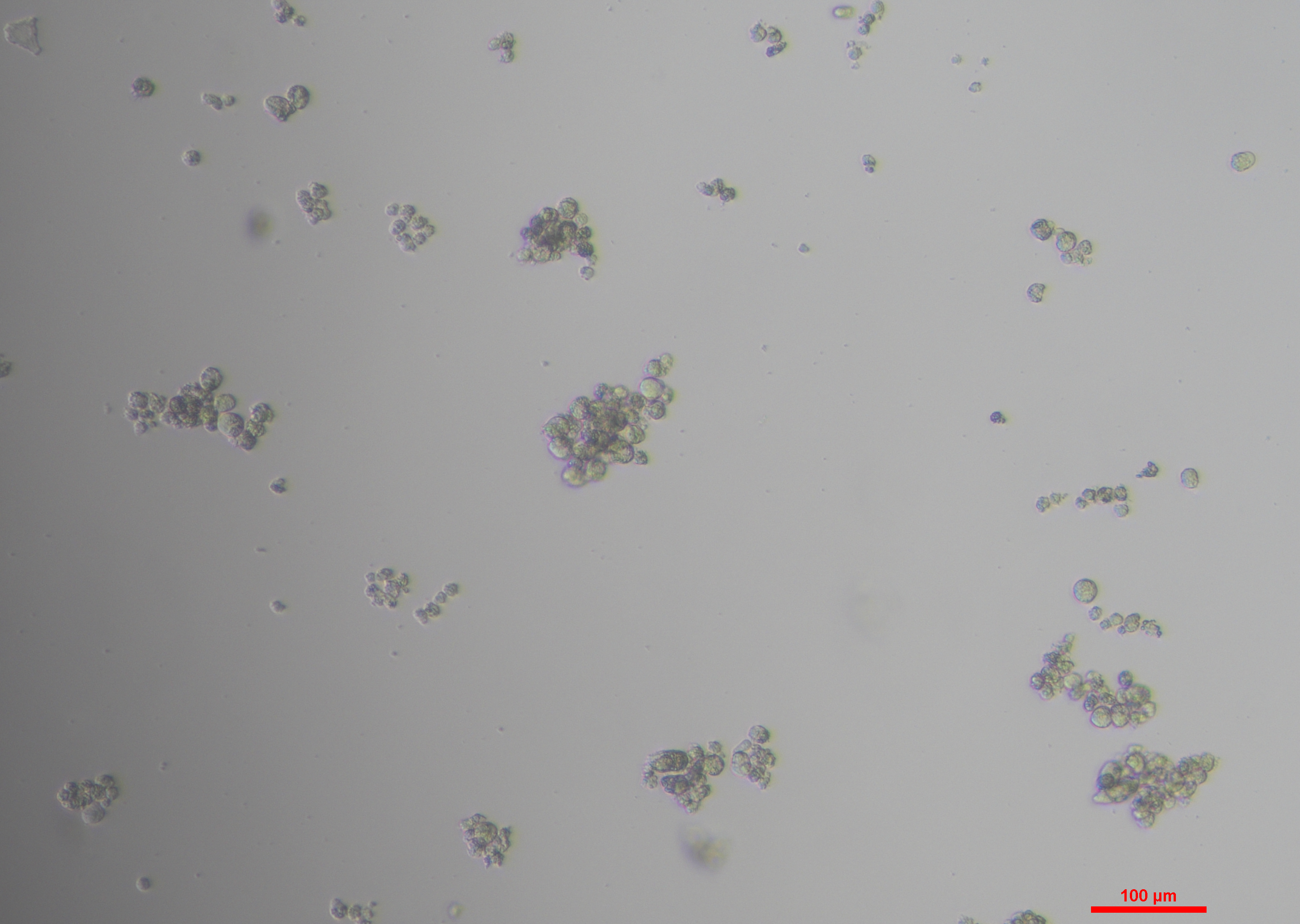

Supplement: Figure 7—source data 1. [file elife-65418-fig7-data1.zip › elife-65418-fig7-data1-v3/F/231/siRHBDL2.tif]

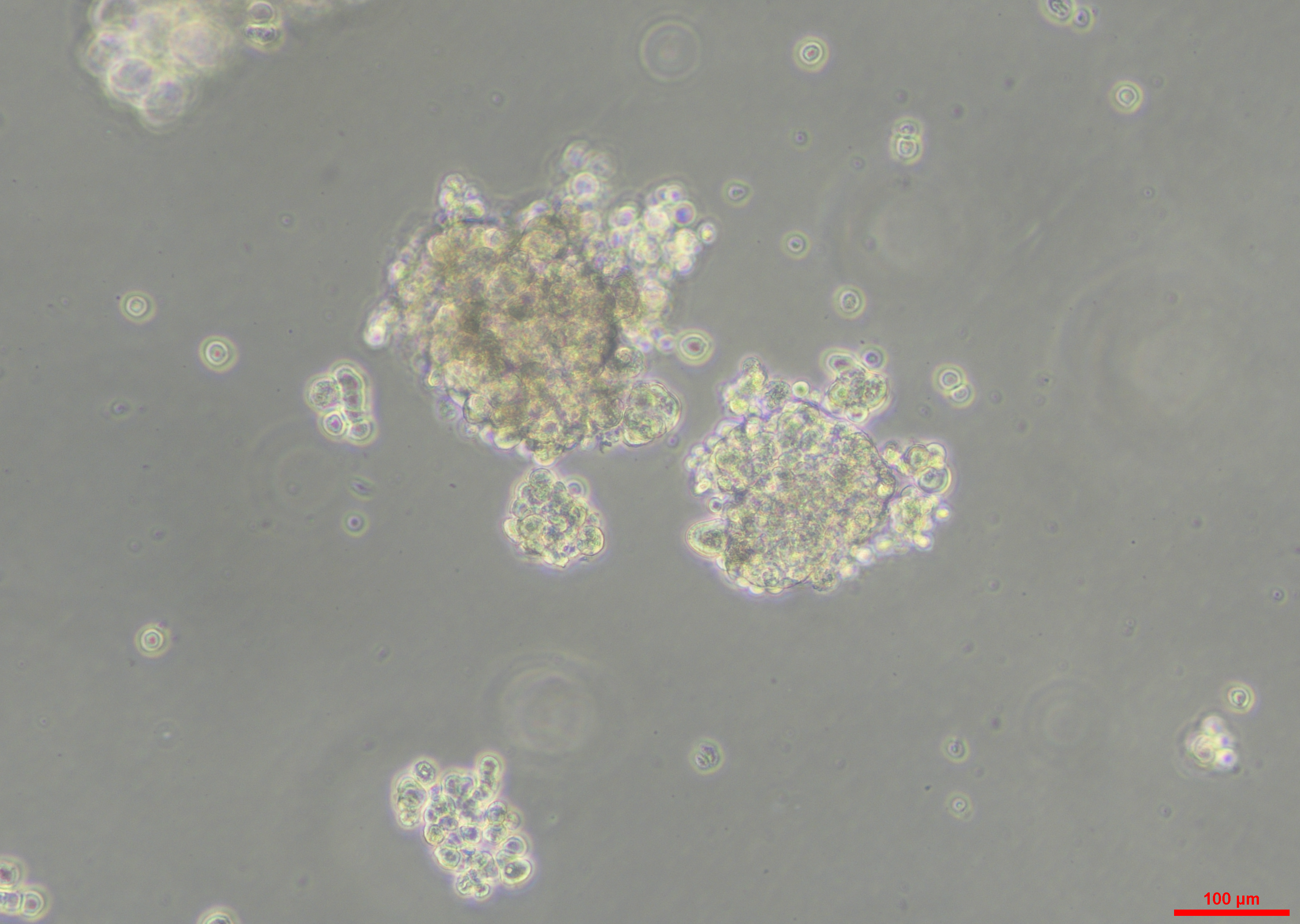

Supplement: Figure 7—source data 1. [file elife-65418-fig7-data1.zip › elife-65418-fig7-data1-v3/F/231/Ctl.tif]

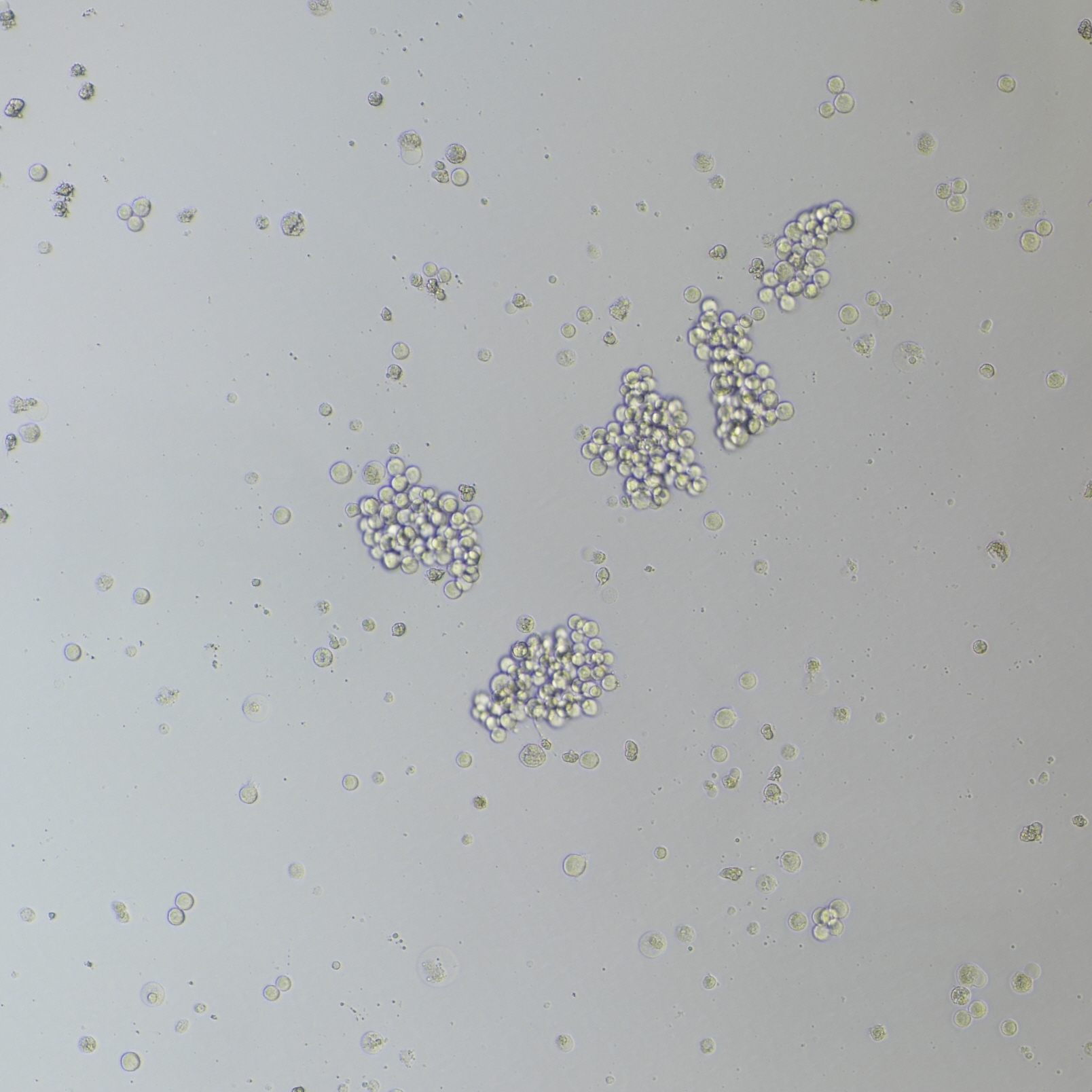

Supplement: Figure 7—source data 1. [file elife-65418-fig7-data1.zip › elife-65418-fig7-data1-v3/F/468/si RHBDL2.jpg]

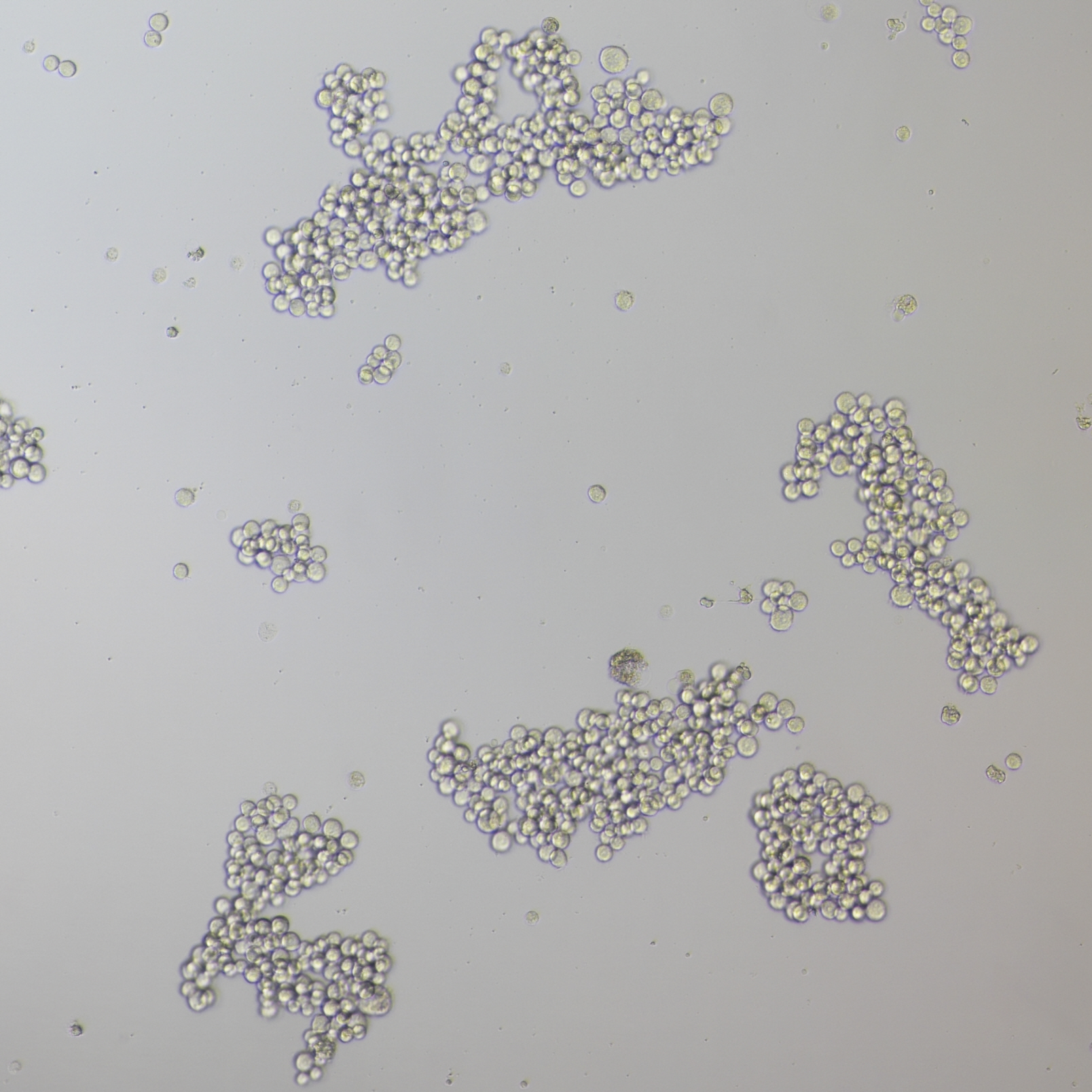

Supplement: Figure 7—source data 1. [file elife-65418-fig7-data1.zip › elife-65418-fig7-data1-v3/F/468/ctl 1.jpg]

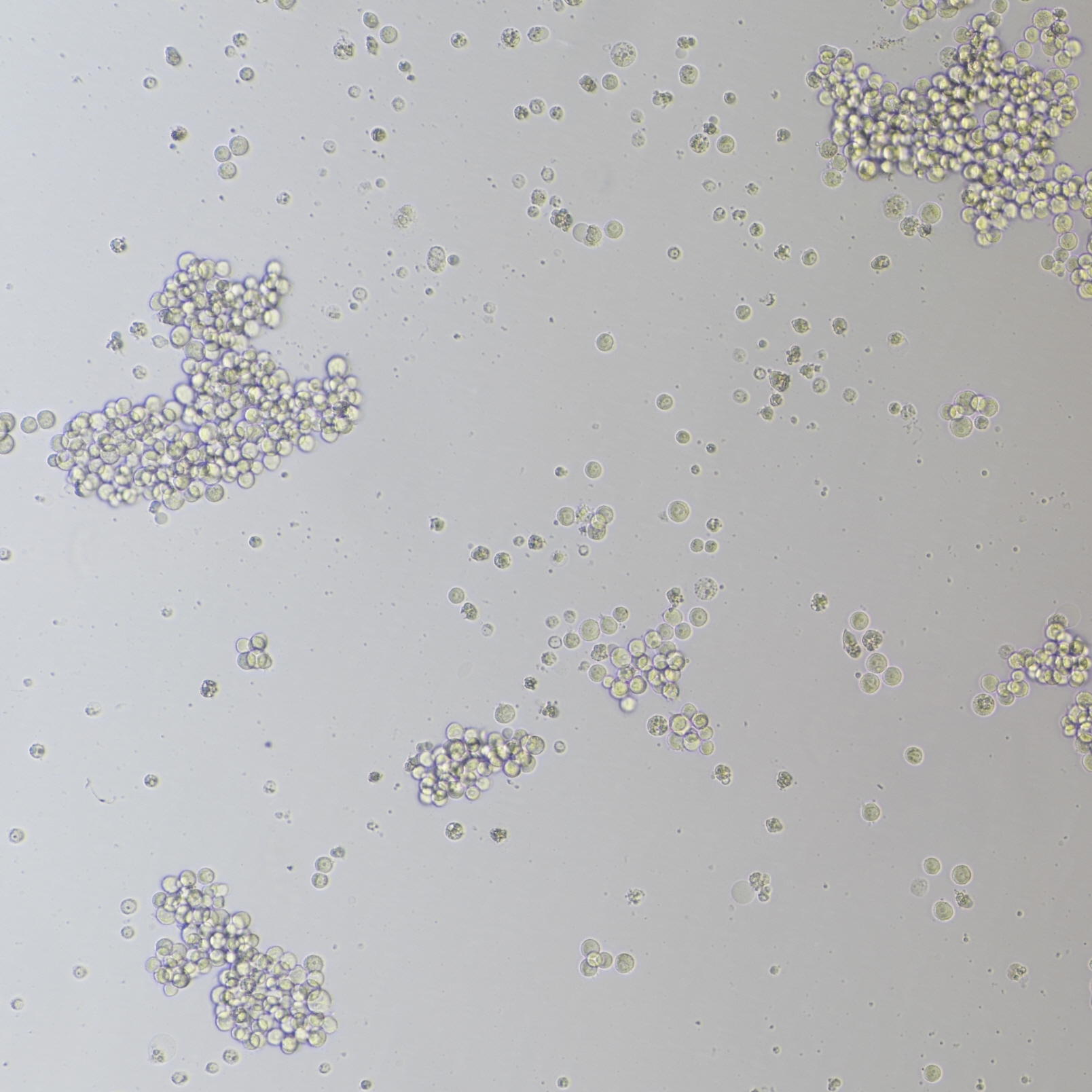

Supplement: Figure 7—source data 1. [file elife-65418-fig7-data1.zip › elife-65418-fig7-data1-v3/F/468/ctl 2.jpg]

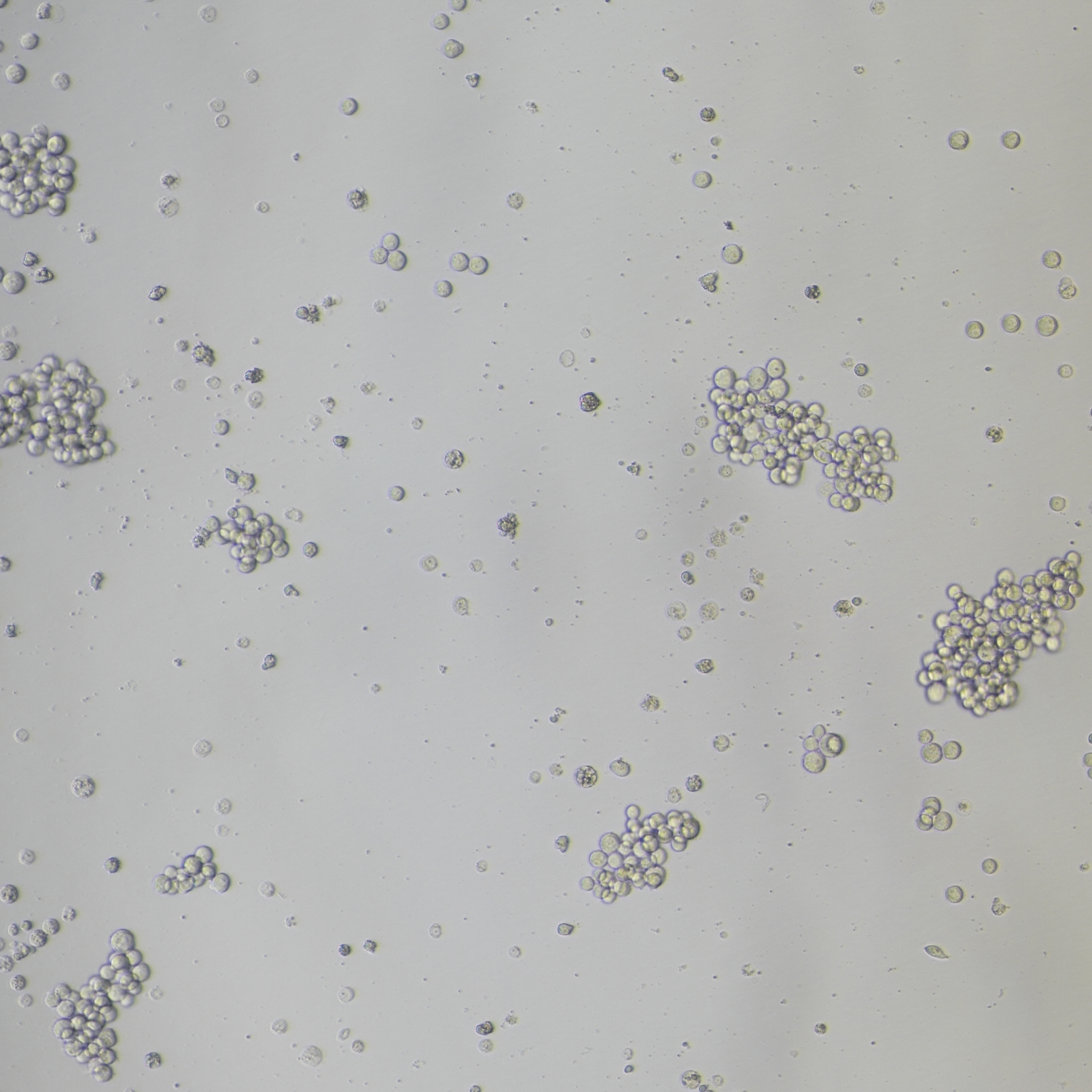

Supplement: Figure 7—source data 1. [file elife-65418-fig7-data1.zip › elife-65418-fig7-data1-v3/F/468/si RHBDL2 2.jpg]
