# Supplementary material for: Association of human breast cancer CD44-/CD24- cells with delayed distant metastasis
Source: eLife. 2021 Jul 28;10:e65418. doi: 10.7554/eLife.65418 (PMC8346282; doi:10.7554/eLife.65418)
Supplement: Supplementary file 1. [file elife-65418-supp1.docx]

**Supplement File 1.** Clinicopathological characteristics of patients

| Variables | Training set n (%) | Testing set n (%) |
| --- | --- | --- |
| Number of patients | 355 | 221 |
| Median age at diagnosis (yrs.) | 51 | 51 |
| Molecular subtype |  |  |
| Luminal | 159 (44.79) | 89 (40.27) |
| HER-2 | 39 (10.99) | 68 (30.77) |
| TNBC | 157 (44.22) | 64 (28.96) |
| N stage |  |  |
| N0 | 240 (67.61) | 123 (55.66) |
| N1 | 69 (19.44) | 62 (28.05) |
| N2 | 25 (7.04) | 17 (7.69) |
| N3 | 21 (5.91) | 19 (8.60) |
| T stage |  |  |
| T1 | 196 (55.21) | 63 (28.51) |
| T2 | 156 (43.94) | 147 (66.51) |
| T3 | 3 (0.85) | 11 (4.98) |
| Clinical stage |  |  |
| I | 110 (30.99) | 31 (14.03) |
| II | 198 (55.77) | 156 (70.59) |
| III | 47 (13.24) | 34 (15.38) |
| Lymph node involvement | 115 (32.39) | 98 (44.34) |
| Distant metastases | 105 (29.58) | 52 (23.53) |
| Bone metastases | 19 (5.35) | 22 (9.95) |
| Follow-up in months | 96 (5 - 96) | 78 (9 - 96) |
| Death | 37 (10.42) | 54 (24.43) |
