# Supplementary material for: Association of human breast cancer CD44-/CD24- cells with delayed distant metastasis
Source: eLife. 2021 Jul 28;10:e65418. doi: 10.7554/eLife.65418 (PMC8346282; doi:10.7554/eLife.65418)
Supplement: Supplementary file 3. [file elife-65418-supp3.docx]

**Supplement File 3.** Univariate and multivariate Cox regression analyses of clinicopathological factors as predictors of DFS

| Variables | Univariate analysis | |  | Multivariate analysis | |
| --- | --- | --- | --- | --- | --- |
|  | HR (95%CI) | *P*-value |  | HR (95%CI) | *P*-value |
| Percentage of CD44^-^/CD24^-^ cells  >19.5% versus <19.5% | 4.153 (2.727–6.326) | **<0.0001** |  | 2.621 (1.631–4.214) | **<0.0001** |
| Frequency of CD44+/CD24- cells  ≥2% versus <2% | 5.259 (3.548–7.794) | **<0.0001** |  | 3.712 (2.384–5.780) | **<0.0001** |
| Lymph node involvement  Yes versus No | 1.672 (1.133–2.468) | **0.0096** |  | 0.322 (0.053–1.943) | 0.21 |
| N stage |  | **0.0023** |  |  | **0.027** |
| N1 versus N0 | 1.510 (0.948–2.405) | 0.083 |  | 3.693 (0.589–23.141) | 0.16 |
| N2 versus N0 | 1.310 (0.599–2.864) | 0.49 |  | 2.437 (0.292–20.316) | 0.41 |
| N3 versus N0 | 3.776 (2.031–7.019) | **<0.0001** |  | 10.166 (1.066–96.998) | **0.043** |
| Molecular subtype |  | **0.025** |  |  | **0.020** |
| HER-2 versus Luminal | 1.296 (0.658–2.553) | 0.45 |  |  |  |
| TNBC versus Luminal | 1.762 (1.160–2.677) | **0.0079** |  | 4.190 (1.244–4.106) | **0.020** |
| Clinical stage |  | **0.0094** |  |  | 0.90 |
| II vs. I | 1.414 (0.887–2.255) | 0.145 |  | 1.065 (0.544–2.085) | 0.85 |
| III vs. I | 2.581 (1.433–4.649) | **0.0016** |  | 1.358 (0.358–5.157) | 0.6526 |
| Ki67 index |  | **0.020** |  |  | 0.16 |
| 14%-30% versus 0-14% | 1.765 (0.813–3.832) | 0.15 |  | 1.760 (0.769–4.029) | 0.18 |
| >30% versus 0-14% | 1.904 (1.201–3.017) | **0.0061** |  | 1.520 (0.911–2.536) | 0.10 |
| PR status  Positive versus Negative | 0.595 (0.395–0.896) | **0.012** |  | 0.801 (0.372–1.723) | 0.56 |
