# Supplementary material for: Association of human breast cancer CD44-/CD24- cells with delayed distant metastasis
Source: eLife. 2021 Jul 28;10:e65418. doi: 10.7554/eLife.65418 (PMC8346282; doi:10.7554/eLife.65418)
Supplement: Supplementary file 4. [file elife-65418-supp4.docx]

**Supplement File 4.** Primary antibodies used in western blotting

| Protein | Catalog number | Manufacturer |
| --- | --- | --- |
| GAPDH  RHBDL2 | 10494-1-AP  12467-1-AP | Proteintech (Rosemont, IL, USA)  Proteintech |
| OCT4 | 11263-1-AP | Proteintech |
| SOX2 | 11064-1-AP | Proteintech |
| ABCB1 | [22336-1-AP](http://www.ptgcn.com/products/ABCB1-Antibody-22336-1-AP.htm) | Proteintech |
| YAP1 | 13584-1-AP | Proteintech |
| Tubulin-β | 10094-1-AP | Proteintech |
| Alpha-tubulin | 11224-1-AP | Proteintech |
| Phospho-YAP | KA1392C | Immunoway (Plano, TX, USA) |
| NF-κB | YM3111 | Immunoway |
| Phospho-NF-κB | YP0188 | Immunoway |
| USP31 | ab240543 | Abcam (Cambridge, MA, USA) |
| NANOG | ab80892 | Abcam |
| Nestin | ab22035 | Abcam |
| Lamin B1 | 12987-1-AP | Proteintech |
